# Magnetic resonance imaging findings in chronic anterior cruciate ligament‐deficient knees: A systematic review of meniscal, cartilage, tibial translation and ligamentous changes

**DOI:** 10.1002/jeo2.70696

**Published:** 2026-03-24

**Authors:** Abel Davtyan, Ajay Shah, Quang Le, Ananya Pathak, Veronika Kolarska, Praveen Sritharan, Paul Marks, David Wasserstein

**Affiliations:** ^1^ School of Medicine University of Limerick Limerick Ireland; ^2^ Schatzker Joint Preservation Initiative, Sunnybrook Health Sciences Centre University of Toronto Toronto Ontario Canada; ^3^ Division of Orthopaedic Surgery, Women's College Hospital Toronto Ontario Canada; ^4^ Faculty of Arts and Science University of Toronto Toronto Ontario Canada; ^5^ Tanenbaum Institute for Science in Sport Toronto Ontario Canada

**Keywords:** acute cruciate ligament, chronic, knee, MRI, systematic review

## Abstract

**Purpose:**

The purpose of this study is to systematically review the literature for time‐dependent changes in knee anatomy and morphology on magnetic resonance imaging (MRI) in patients with chronically deficient anterior cruciate ligament (ACL) knees.

**Methods:**

Four databases were searched from inception until 19 January 2025, for primary research studies (level of evidence: II–IV) reporting MRI findings in chronically (defined as >6 months) ACL‐deficient patients. Data regarding time from injury to MRI, sample size, demographics (age, biological sex), comparison group, and MRI findings (meniscal tears, cartilage injuries, posterior cruciate ligament [PCL] angles, collateral injuries, anterior tibial subluxation) were abstracted; cohort studies with acute ACL tear groups were included for pooled comparison. Data were presented narratively and pooled for statistical analysis when possible.

**Results:**

From the 22 studies selected, 2863 patients were included, mean age was 27.9 years (range: 22.6–35.0, ±2.91 years), and 1957 were males (68.3%). A total of 1360 patients had chronically deficient ACL knees (>6 months from injury). The weighted mean time from injury to MRI was 27.2 months (range: 12.8–66.0 months, 11 studies). Compared to acute ACL‐injured knees, chronic anterior cruciate ligament deficiency (ACLD) patients had a higher rate of medial meniscus injuries (50.3% vs. 36.0%, *p* < 0.001), medial femoral condyle lesions, medial tibial plateau cartilage lesions, and anterior medial tibial subluxation (+1.6 mm, *p* < 0.001). The incidence of medial meniscus injuries increased linearly over time. Pooled analysis showed no differences between acute and chronic ACLD knees for lateral meniscus injuries or anterior lateral tibial subluxation. Chronic ACL‐deficient knees demonstrated greater PCL buckling, anterolateral ligament attenuation, ACL fragmentation, alongside reduced bone bruising and joint effusion.

**Conclusions:**

This systematic review found a higher rate of medial meniscus injuries, medial femoral condyle and medial tibial plateau cartilage lesions, anterior medial tibial subluxation, PCL verticality and ALL and ACL attenuation in chronic ACL‐deficient knees.

**Level of Evidence:**

Level II–IV, systematic review.

## Graphical abstract

This systematic review of 22 MRI studies (*n* = 2863) demonstrates that chronic ACL‐deficient knees exhibit significantly higher rates of medial meniscal tears, cartilage lesions, anterior medial tibial subluxation, and secondary ligamentous changes compared to acute ACL injuries.

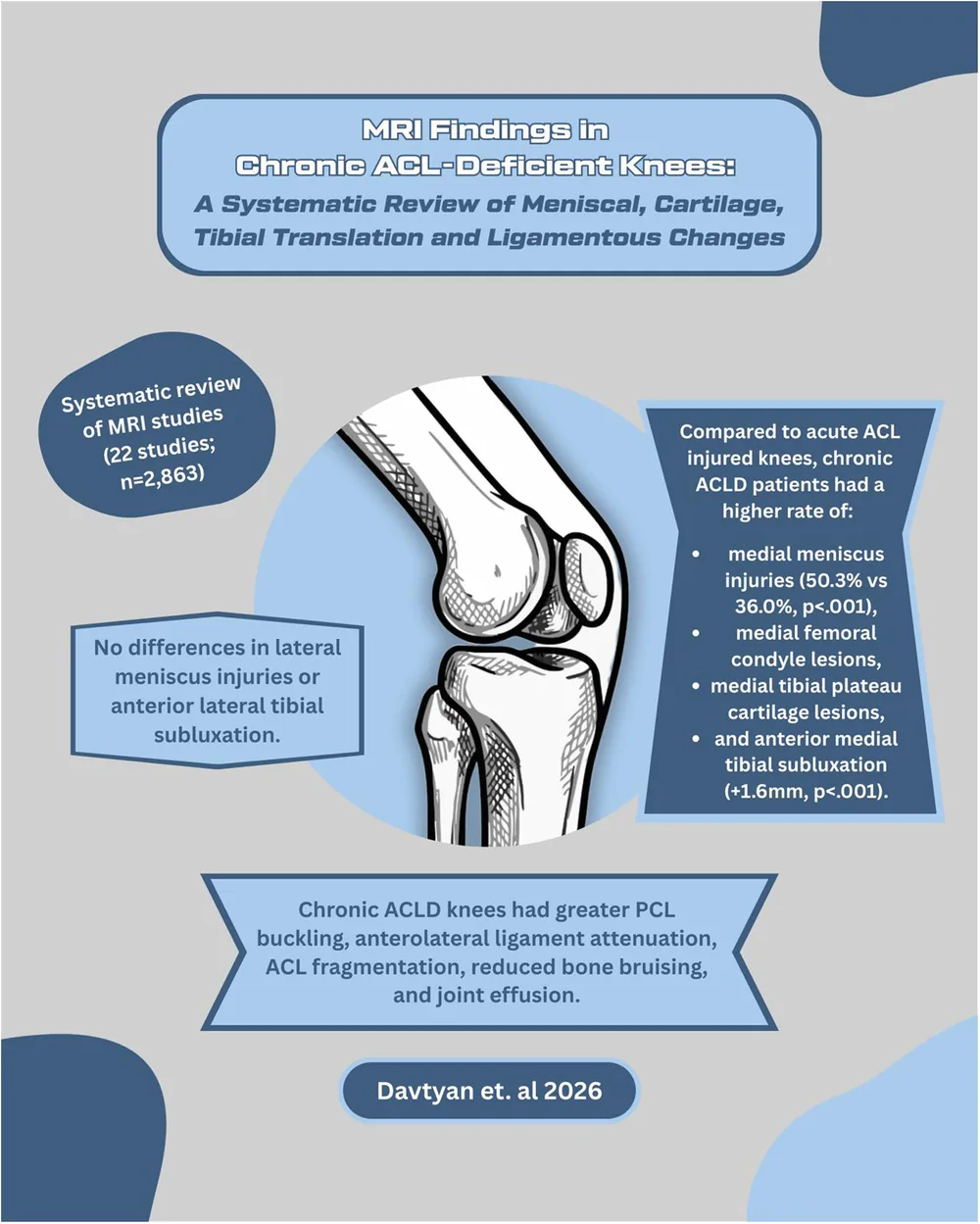

**Abbreviations**
ACL: anterior cruciate ligament
ACLD: anterior cruciate ligament deficiency
ACLR: anterior cruciate ligament reconstruction
ACLT: anterior cruciate ligament tear
ALL: anterior‐lateral ligament
LCL: lateral cruciate ligament
LFC: lateral femoral cartilage
LM: lateral meniscus
MCL: medial cruciate ligament
MFC: medial femoral cartilage
MM: medial meniscus
MRI: magnetic resonance imaging
OR: odds ratio
PCL: posterior cruciate ligament
RCT: randomized control trial
TFI: time from injury

## INTRODUCTION

Injuries to the anterior cruciate ligament (ACL) are among the most common and serious knee injuries, and they frequently result in long‑term disability, reduced quality of life and chronic knee instability across athletic and non‑athletic populations [16]. European data, specifically from Sweden, shows a population level incidence rate of 78 per 100,000 persons between 2001 and 2009 [35]. Furthermore, MRI‐diagnosed ACL ruptures portray a similar incidence rate of 75.1 per 100,000 persons in a Swedish cohort [33]. Population‐based data estimate that the annual incidence of isolated ACL tears in Canada is approximately 68.6 per 100,000 person‑years, with a peak incidence of 241 per 100,000 in males aged 19–25 and 227 per 100,000 in females aged 14–18 [9, 24, 38, 59]. A 10‐year retrospective cohort from the United States showed an incidence of 931,186 ACL tears, with 734,597 (78.8%) being treated non‐operatively [3].

Non‐operative treatment of ACL injury is an option within the standard of care, with several RCTs of ACL reconstruction (ACLR) versus functional rehabilitation demonstrating minimal differences in return‐to‐sport or activity level [10]. Purposeful delayed ACLR has become part of the treatment algorithm in those who fail functional rehabilitation, as demonstrated by the Delaware–Oslo and KANON trials [26, 43]. Long‐term ACL deficiency can also occur due to barriers in care and other factors that delay surgical management and assessment, such as misdiagnosis, lack of healthcare insurance or access and minimal injury symptoms.

Chronically deficient ACL (ACLD) knees appear to undergo several anatomical and biomechanical changes, which may change outcomes compared to knees without these changes. Studies posit biomechanical changes such as anterior tibial translation and increased posterior cruciate ligament (PCL) angle [7]. Other studies indicate changes such as meniscus and chondral damage, and changes in joint morphology [42, 49]. Although most studies currently stratify the ACL‐deficient knee by chronicity, secondary structural changes may play a more important role from a treatment and prognostic perspective. While meniscal and cartilage changes in the chronic versus acute ACL‐deficient knee have been well defined in the literature [12, 18], less is known about secondary morphological changes, such as ligament appearances and tibial translation.

The purpose of this study is to systematically review the literature for time‐dependent changes in knee anatomy and morphology on magnetic resonance imaging (MRI) in patients with chronically deficient ACL knees that did not undergo ACLR. In doing so, this study hopes to better understand the natural history of the patient with the chronically ACL‐deficient knee and provide patients and clinicians with more information in clinical decision‐making. The hypothesis of this study is that time‐dependent changes will be seen in meniscal, chondral, and alignment, particularly within the medial tibiofemoral compartment.

## METHODS

### Search strategy

Four online databases (EMBASE, Ovid MEDLINE, CINAHL and CENTRAL) were searched for literature from database inception until 19 January 2025, investigating the MRI findings in chronically deficient ACL knees. The search included the terms: anterior cruciate ligament, MRI, and deficiency (full search strategy is in Supporting Information S1: Table S4). Hand‐searching and snowball searching of references of included studies and were employed to identify grey literature and unpublished or conference abstracts, searches were updated manually over the course of the year to confirm new publications. This study was registered to PROSPERO under the code: CRD420261282572).

### Study screening

The titles, abstracts, and full‐text articles were screened by four reviewers independently and in duplicate. Disagreements during title and abstract screening moved into the next stage, for an in‐depth review. Any disagreements were discussed between reviewers, and a senior author was consulted. The references of the included studies were subsequently manually screened for additional articles that may have eluded the initial search strategy.

### Assessment of study eligibility

The research question and study eligibility criteria were established a priori. The inclusion criteria were studies investigating humans, studies with levels of evidence I to IV, and those reporting MRI‐based findings in chronically ACL‐deficient knees (>6 months since injury). Exclusion criteria were animal studies, commentaries, book chapters, review articles, conference abstracts and technical studies. Authors with incomplete datasets were contacted via e‐mail to request additional data when required.

### Data extraction

Data were collected by four reviewers and recorded in a Microsoft Excel spreadsheet (Version 2007, Microsoft). Abstracted data included the authors, year of publication, study design, sample sizes, sex ratio, mean age, comparator groups, time from injury to MRI, time to surgery, ACL appearance, meniscal pathologies, cartilage pathologies, ligamentous pathology, anterior tibial subluxation, tibial internal rotation, joint effusion, PCL angle, posterior tibial slope, bone bruising and bony anatomy.

### Quality assessment

The methodological quality of the included studies was assessed using the MINORS instrument [41]. This tool was designed to assess the methodological quality of comparative and non‐comparative, observational non‐randomised studies. A table depicting the scoring of each included article can be found in the supplementary files (Supporting Information S1: Table S1).

### Assessment of agreement

To assess the inter‐reviewer agreement, a kappa (*κ*) statistic was calculated for the title, abstract, and full‐text screening stages. An intra‐class correlation coefficient (ICC) was calculated for the quality assessment using the MINORS criteria. Agreement was categorised a priori as follows: *κ*/ICC of 0.61 or greater was considered substantial agreement; *κ*/ICC of 0.21–0.60, moderate agreement; and *κ*/ICC of 0.20 or less indicating slight agreement.

### Statistical analysis

Descriptive statistics, including means, proportions, standard deviations, and 95% confidence intervals (CIs) were calculated using Minitab® statistical software (Version 17, Minitab Inc.). Outcomes such as meniscus tears (medial/lateral), chondral injuries, and tibial translation were extracted from comparative studies as binary variables and pooled for meta‐analysis when possible. Meta‐analysis was performed using the RevMan Software (Cochrane Collaboration), using the Mantel–Haenszel (M–H) ratio with random effects, heterogeneity was reported. The prevalence of meniscal tears and follow‐up time were plotted using a scatter plot and fitted with a line of best fit using the Akaike Information Criterion. Other outcomes were heterogenous and the results are presented narratively.

## RESULTS

### Search results

The initial search of the online databases resulted in 1421 total studies. A systematic screening and assessment of eligibility identified 22 full‐text articles that satisfied the inclusion and exclusion criteria (Figure 1). Search terms can be found in Supporting Information S1: Table S4. All MRI findings were performed pre‐operatively and on non‐operative knees. The reviewers reached substantial agreement at the title and abstract stage (*κ* = 0.894; 95% CI: 0.865–0.923), the full‐text stage (*κ* = 0.847; 95% CI: 0.764–0.930), and final stage (*κ* = 1.00).

**Figure 1:**
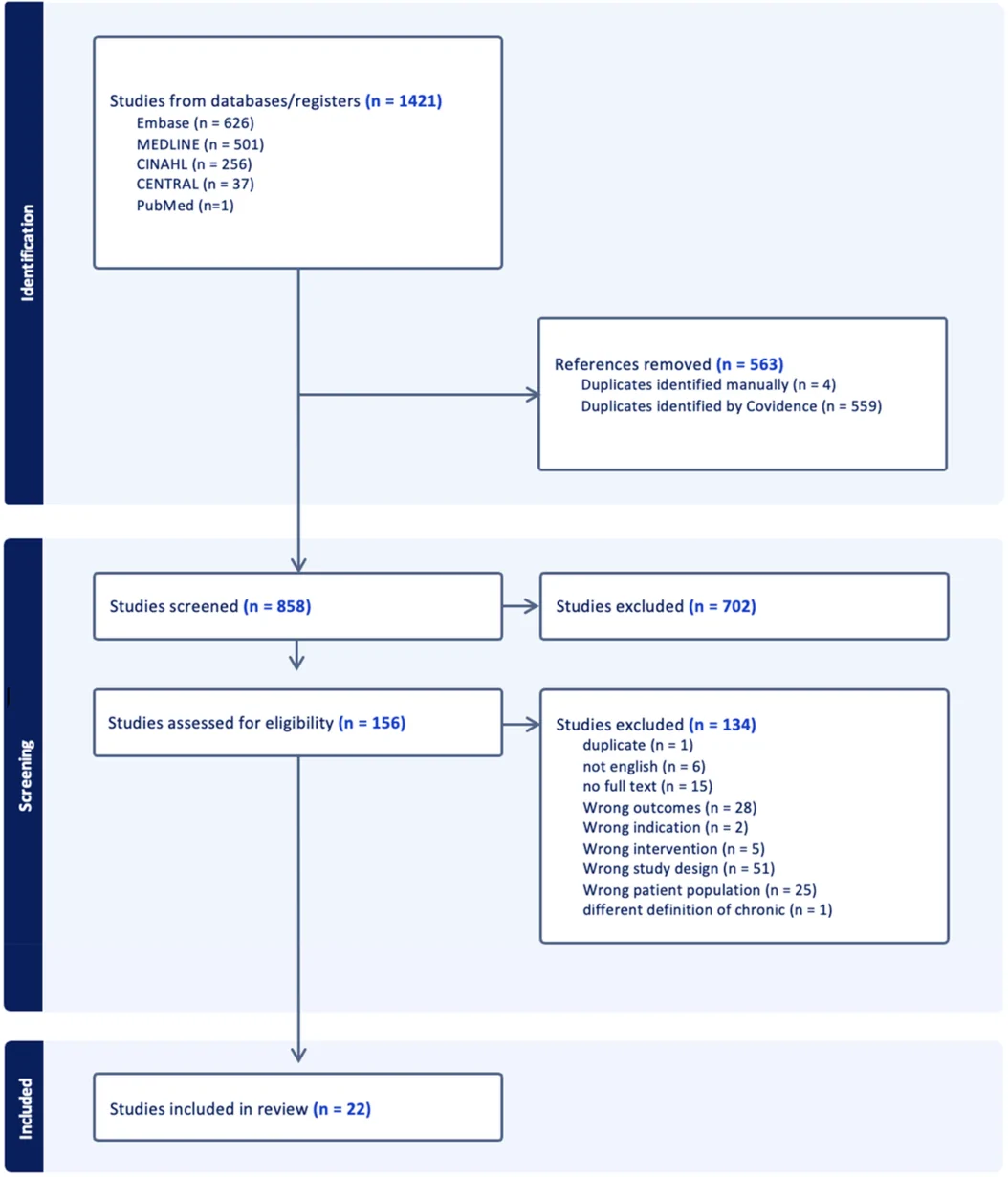
PRISMA Flow diagram demonstrating the systematic review of the literature for chronic anterior cruciate ligament (ACL) deficiency: magnetic resonance imaging (MRI) findings.

### Study quality and characteristics

Of the 22 studies included, 13 were retrospective studies [2, 8, 13, 25, 30, 31, 34, 36, 47, 52, 56, 57, 60], 4 were prospective studies [6, 48, 49, 58], 4 were cross‐sectional studies [29, 37, 50, 53] and 1 was a case series [54]. All studies were comparative, apart from Vlychou et al. [49], Yoo et al. [54] and Mishima [30]. Eight of the 22 studies were cohort‐matched for age and sex [2, 13, 25, 34, 50, 52, 53, 60]. Table 1 depicts all study characteristics. The mean MINORS score for all studies was 19.9 (ranging from 16 to 23), corresponding to a moderate‐high level of methodological reporting quality. The MINORS table is available in the supplemental files (Supporting Information S1: Table S1).

**Table 1:** Study characteristics.

Author	Year	Level of evidence, study type	Sample size	Mean age	Sex (male [%])	Sample size chronic ACLD (male)	Definition of chronic ACL injury	Control group[s] criteria/definition	Time from injury to MRI (mean/median, range)	Time from injury to surgery (mean/median, range)
Dimond et al.	1998	3/Retrospective comparative study	87	28.9	50 (57.5)	27 (18)	More than 6 months after injury	Acute ACL tear	55.4 weeks (26.4 weeks to 9.9 years)	NA
Vahey et al.	1991	3/Retrospective evaluation	79 (81 knees)	32	50 (63.2)	30 (NR)	>6 months post‐injury	Acute ACL tear	5.5 years (6 months to 21 years)	NA
Zeng et al.	2024	3/Prospective comparative cohort study	65	28.7	51 (78.5)	30 (26)	12–36 months post‐injury	Healthy controls	ACLD with CL: 12.7 ± 1.4 months, ACLD without CL: 13.6 ± 1.5 months	NA
Crook et al.	2021	4/Prospective observational case series	8	35	6 (75)	8 (6)	>6 months post‐injury	Contralateral uninjured knee	Mean time = 3.67 years, range = 87 days to 11 years.	NA
Ates and Ogul	2024	4/Retrospective cohort study	100	28.75 ± 8.45	94 (94)	61 (56)	>6 months post‐injury, 3 sub‐ groups: (i) 7–12, (ii) 13–24, (iii) >24 months	Acute ACLt	MRIs must have been taken within 30 days of the surgery, no exact average time to surgery or MRI given	NA
McDonald et al.	2017	3/Cross‐sectional study	212	25.2 (ACL) 37.1 (control)	118 (55.7)	40 (24)	>12 months post‐injury	Healthy controls	NR	Chronic group: 3119 ± 3632 days. (range 349–12,915)
Osaki et al.	2015	3/Cross‐sectional study	63	25.2 ± 8.6 (ACL injuries), 37.1 ± 6.0 (healthy control)	44 (69.8%)	Group B: 14 (10), Group C: 7 (4)	Group C: ≥2 years post‑injury, Group B: (semi‐chronic group) >12 weeks post‐injury	Healthy controls	Group B: 256.3 ± 171.2 days (range 97–551) Group C: 1328.7 ± 397.2 (744–1738)	NA
Yoon et al.	2010	3/Retrospective diagnostic study	145	31.3 ± 10.2	124 (85.5)	Semi‐Chronic Group: 23 (21) Chronic Group: 36 (32)	>1‐year post‐injury (semi‐chronic group 3‐12 months post‐injury)	Acute ACL tears: <6 weeks post‐injury, Subacute ACL tears: 6 weeks to 3 months post‐injury	2166.3 ± 1599.6	NA
Nawata et al.	1993	3/Retrospective comparative cohort study	56	27.8	26 (46.4%)	20	>12 months post‐injury	Acute ACL rupture (<1‐month post‐injury), Subacute ACL rupture (1–12 months post‐injury)	NR	NR
Yoon et al.	2013	3/Retrospective diagnostic study	145	31.3 ± 10.2	108 (74.5)	38 (NR)	>12 months post‐injury	N/A	2,220.5 (±1691.8) in chronic group	N/A
Liu et al.	2022	3/Retrospective prognostic case‐control study	90	24.7	69 (77)	52	3 months post injury, however, cohort was all chronic for at least 1 year	Age‐ and sex‐matched patients, < 3 months of chronicity (Acute ACLt)	High Grade chronic group (*n* = 11): 27.9 (11.9–43.9) months Low‐grade chronic group (*n* = 41): 12.4 (9.6–15.1) months	NA
Ye et al.	2023	3/cross‐sectional study	226	29.2	160 (70.8)	124	Defined at 3+ months of deficiency. Entire cohort mean TFI is 1+ years	Acute ACLt, less than 3 months following injury (*n* = 102)	NR	Mean 22.8 months (high‐grade); 11.9 months (low‐grade)
Wang et al.	2021	3/Cross‐sectional observational study	272	29.6	166 (61.1)	48 (CR2: 30 (24), CR3: 18 (13))	CR2: 1–5 years, CR3: 5+ years	Healthy controls	NR	CR2: 1–5 years, CR3: >5 years
Vlychou et al.	2011	2/Prospective diagnostic study	43	22.6	39 (90.7)	43 (39)	ACLD > 1.5 years	NA	24–48 h prior to surgery (19.6 months)	19.6 months
Xu et al.	2018	3/Retrospective comparative cohort	377	NR	274 (72.7)	217 (157)	Chronic defined at 6 weeks, with average of 1.3 years TFI	Acute ACLt with ACLR	Acute: 3.2 weeks (range: 1–47 days), Chronic: 1.3 years (range: 11 weeks to 10 years)	NR
van Meer et al.	2016	2/Prospective cohort study	143	25.2 (21.4–32.6)	94 (65.7)	43	>6 months post‐injury	ACLR < 6 months, *n* = 50 Intact ACL, *n* = 50	MRI at 1 month post injury, and MRI at 24 months posts injury, Patients categorised into non‐reconstruction and reconstruction groups within those 2 years	5.5 (3.3–8.9) Median and IQR. 50 patients received reconstruction before 6 months, 43 received after 6 months, 50 were non‐operative
Yoo et al.	2009	4/Case‐series	31	28.5 (8.5)	27 (87.1)	31	NR	N/A	First MRI: 8.5 months. Second MRI: 46.9 months	49.9 months (range, 12–132 months).
Nonaka et al.	2024	3/Retrospective cohort study	50	26.6 (11.5 years)	29 (48.0)	50	NR	Acute ACLRs with no LM or other lesions	NR	Gap positive group: 1,121.3 (3089.5) d, Gap‐Negative group: 329.9 (778.9)
Oronowicz et al.	2023	3/Retrospective cohort study	80	25.6 (19.7–34.0)	48 (60.0)	22	>6 months post‐injury	Acute ACLR	0.7 (0.2–119.3)	12.9 months (8.1–44.4)
Gong et al.	2023	3/Retrospective cohort study	433	30.1	308 (71.1)	157	>6 months post‐injury	Acute ACL tears: <6 weeks, Subacute ACL tears: 6 weeks to 6 months	NR	NR
Mishima et al.	2005	3/Retrospective case series	96	23.8 years (range, 14 to 48 years)	50 (52%)	96	NR	Healthy contralateral knee	27 months (range, 1 month to 25 years)	NR

Abbreviations: ACL, anterior cruciate ligament; ACLD, anterior cruciate ligament deficiency; ACLR, anterior cruciate ligament reconstruction; ACLt, anterior cruciate ligament tear; LM, lateral meniscus; MRI, magnetic resonance imaging; NR, not reported.

In total, 2863 patients were included, mean age was 27.9 years (range: 22.6–35.0, ±2.91 years), and 1957 were males (68.3%). A total of 1360 patients had chronically deficient ACL knees (>6 months from injury). The weighted mean time from injury to MRI was 27.2 months (range: 12.8–66.0 months, 11 studies). Several comparator groups were used: healthy controls, healthy contralateral knees, acute ACLD (<3 months) and subacute ACLD (3–6 months). Key Findings of this study are summarised in Table 2. For a full table of results for all MRI findings, please refer to Supporting Information S1: Table S2. Table 3 provides term definitions within each study, as there is heterogenous definitions of concepts across studies.

**Table 2:** Key findings.

Author	Key findings
Dimond et al.	Chronic ACL tear patients show higher rates of medial meniscal tears (78% vs. 40%, *p* = 0.03), articular cartilage damage (19% vs. 0%), and greater PCL bowing ratio (0.47 vs. 0.37, *p* = 0.013) compared to acute; bone bruises only in acute (68% vs. 0%, *p* = 0.001).
Vahey et al.	Fibrous scarring within the intercondylar notch is a key finding in chronic tears.
Zeng et al.	Cartilage lesions were seen in 18/30 chronic ACLD knees. Lesions were primarily in the posterior medial tibial plateau (17/18), posterior lateral tibial plateau (16/18; grade III; *n* = 5), and anterior medial femoral condyle (16/18). 44/72 patients developed secondary meniscal pathology during the study period.
Crook et al.	Cartilage strain was significantly higher in ACL‐deficient knees at medial intercondylar notch (6% vs. 2%, *p* = 0.01) and medial/lateral tibial plateau (4% vs. 3%, *p* = 0.01).
Ates and Ogul	ALL healing is more common in the acute group (Grade A = 51.3%) versus chronic group (Grade A = 16.4%). Statistically significant difference in ALL grades between acute and chronic (*p* < 0.001) suggests that ALL healing potential decreases with time from ACL injury.
McDonald et al.	Acute ACLD patients (TFI = 1.1 months), presented with a 15% (11/74) rate of medial femoral condyle (MFC) lesions, while chronic ACLD patients (TFI = 102.45 months) presented with 67.5% (27/40) MFC cartilage lesions (*p* = 0.001). Chronic and failed ACL reconstruction knees have significantly greater anterior tibial subluxation than controls. 10/40 (25%) chronic and 27/72 (37.5%) failed ACLs showed ≥6 mm lateral subluxation, compared to 0 in healthy controls (*p* < 0.0004). ALTS Significant differences were found between the intact and chronic ACL groups (*p* < 0.0001). Combined medial and lateral meniscal tears increase lateral subluxation (*p*= 0.0022). ALL injuries associated with greater subluxation (not independently predictive).
Osaki et al.	Chronic ACL injuries have more cartilage strain in the anterior MFC: 50.6 ± 8.0 ms versus controls 43.9 ± 3.3 ms (*p* < 0.01), and middle MFC: 52.4 ± 7.4 ms versus controls 44.8 ± 2.2 ms (*p* < 0.001). Medial meniscus tears worsen cartilage strain in the anterior MFC: 49.2 ± 7.4 ms versus 45.0 ± 2.8 ms (*p* = 0.03) and middle MFC: 51.5 ± 6.5 ms versus 46.0 ± 1.7 ms (*p* < 0.001). ACL deficient knees have increased risk of cartilage degeneration in the medial femoral condyle beyond 2 years post‑injury.
Yoon et al.	ACL type 1 morphology is dominant in acute knees, while type 3 is dominant in chronic knees. A strong correlation between ACL injury chronicity and PCL angle was observed (*r* = −0.56).
Nawata et al.	Bone bruises most frequently occur acutely; LFC + LTP most affected; bruising fades over time (*p* < 0.01).
Yoon et al.	PCL angle decreased with chronicity of injury (Spearman coefficient, −0.567; *p* < 0.001). High grade ACL morphology was more prevalent in older tears (*p* < 0.001).
Liu et al.	Across the acute and chronic groups, ALTS was higher in the low‐grade chronic group (6.7, range: 5.6–7.8) compared to the acute low‐grade group (4.3, range: 2.9–5.8). AMTS was also higher in both the high and low grade chronic groups (5.1 and 3.9) compared to the acute group (2.7 and 1.8). Chronic ACLD patients had a higher value for lateral tibial posterior slope (7.3 and 7.2 degrees) compared to the acute patients at 6.7 (4.9–8.5) degrees. High‐grade anterior tibial subluxation of the lateral compartment is an independent predictor of high‐grade rotatory instability (odds ratio, 12.992; *p* = 0.011).
Ye et al.	PHLMT was determined to be an independent risk factor for knee laxity (OR, 2.39; 95% CI, 1.20–4.78; *p* = 0.014). High‐grade laxity ACLD patients (TFI = 22.8 months) had a 53/113 (46.9%) rate of posterior horn of medial meniscus tears (PHMMT), while the low‐grade group (TFI = 11.9 months) had a statistically lower PHMMT rate of 30/113 (26.5%) (*p* = 0.002). Optimal cutoff values for predicting high‐grade knee laxity: ATSMC ≥ 2.6 mm, ATSLC ≥ 4.5 mm—ATSLC ≥ 4.5 mm associated with high‐grade knee laxity (OR: 2.94; 95% CI: 1.56–5.55; *p* = 0.001)—AUCs: ATSMC = 0.671, ATSLC = 0.710
Wang et al.	Significant secondary notch stenosis observed over 5 years after ACL rupture. Statistically smaller intercondylar width and notch‐width index in group CR3 compared to other groups (*p* = 0.004, 0.016, and 0.005, respectively).
Vlychou et al.	Medial meniscus tears: 17/43 (39.5%); with 8/17 being complex horizontal tears. Sensitivity and specificity for medial meniscus tears: 93.7% and 92.6%. Lateral meniscus tears: 14/43 (32.6%), with 7/14 being vertical tears. Sensitivity and specificity for lateral meniscus tears: 85.7% and 93.1%. 26 cartilage defects were detected on MRI, 11 were MFC, 11 were LFC, 2 were Patellar cartilage, and 2 were MTC. Moderate agreement for grade I and II cartilage lesions between MRI and arthroscopy; substantial agreement for grade III and IV.
Xu et al.	Diffuse high signal of the ACL on T2‐weighed image was a strong predictor for acute ACL tear rather than chronic ACL tear (55% vs. 3.2%).
van Meer et al.	Medial tibiofemoral compartment cartilage lesions: Reconstruction <6 months OR: 1.17 (0.39–3.52) Reconstruction >6 months OR: 0.30 (0.06–1.53) Lateral tibiofemoral compartment cartilage lesions: Reconstruction <6 months OR: 0.53 (0.21–1.32) Reconstruction >6months OR: 0.82 (0.34–2.01)
Yoo et al.	Medial meniscus, first MRI: 16/31, 2nd MRI: 26/31. Bucket handle tears increased from 2 to 7 from 1st to 2nd MRI. Longitudinal tears increased from 14 to 19.
Nonaka et al.	The gap positive group (TFI = 36.84 months) had a lateral meniscus tear rate of 20/29 (69%), with the tears being subdivided into nine posterior root tears, eight posterior segment tears, two middle segment tears and one anterior segment tear. The gap‐negative group (TFI = 10.84 months) had a lateral meniscus tear rate of 15/21 (71%), with the tears being subdivided into five posterior root tears, nine posterior segment tears and one middle segment tear.
Oronowicz et al.	The median PCL–PCA was significantly lower (−7.7°) in chronic injuries compared to acute injuries (*p* < 0.01), which indicates a higher buckling phenomenon of the PCL in chronic ACL injuries.
Gong et al.	PCL Index for the acute subgroup (*n* = 148) was 5.3 ± 1.7, compared to the subacute group (*n* = 128, PCL index: 5.1 ± 1.6) and the chronic group (*n* = 157, PCL index: 4.8 ± 1.4). There was a significant decrease in the PCLI for the chronic group (*p* < 0.004), representing that the relative advancement and internal rotation of the tibia cause curvature of the PCL.
Zhang et al.	Pre‐operative MRI findings (mean = 13 days prior to reconstruction) showed no significant differences in medial, lateral, global, and internal rotational anterior subluxation. Increased rate of MM injury in chronic ACLd versus acute ACLD (27/31 vs. 18/31).
Mishima et al.	Positive correlation in the extent of the ATS and time since injury (*r* = 0.332, *p* < 0.0013).

Abbreviations: ACL, anterior cruciate ligament; ACLD, anterior cruciate ligament deficiency; ALL, anterior‐lateral ligament; ATSLC, anterior tibial subluxation of lateral compartment; ATSMC, anterior tibial subluxation of medial compartment; CI, confidence interval; LFC, lateral femoral cartilage; LTP, lateral tibial plateau; MFC, medial femoral cartilage; MTC, medial tibial condyle; OR, odd ratio; PCA, posterior condylar axis; PCL, posterior cruciate ligament; PCLI, posterior cruciate ligament index.

**Table 3:** Term definitions by study.

Author	Term	Definition
Dimond et al.	Articular cartilage damage	The contour of the tibiofemoral articular cartilage on proton density‐, T2‐, and T1‐weighted images also was assessed for the presence of the thinning, either focal or diffuse, that extended to the subchondral bone. Identification of abnormalities of the articular cartilage also was based on altered signal intensity within the cartilage.
	PCL bowing sign	The curvature or bowing of the PCL was quantified by a ratio of two measurements: (1) the distance between the anterior most tibial and the femoral insertions of the ligament (line y) and (2) the maximal distance of a perpendicular (line x) drawn between line *y* and the undersurface of the PCL. The PCL bowing ratio is defined as the fractional ratio of the lengths of lines x and y*.*
Zeng et al.	Cartilage lesions	Articular cartilage status was classified as follows: Level 0 for normal cartilage, Level I for signal change without surface defect, Level II for partial‐thickness defect less than 50%, Level III for partial‐thickness defect greater than 50%, and Level IV for full‐thickness defect. The tibiofemoral articular CLs in patients were evaluated based on the modified Noyes Scale.
Crook et al.	Cartilage strain	Cartilage thickness and strain were measured using 3D surface models created from MR images, where strains are defiled as changes in cartilage thickness.
Ates and Ogul	ALL status (Muramatsu Grade)	According to the classification by Muramatsu et al., ALL images on magnetic resonance imaging in the coronal plane: Grade A: Low signal band with clearly defined continuity. Grade B: Ligament showing isosignal change. Grade C: Noncontinuous ALL.
McDonald et al.	Meniscal lesion	Meniscal tears were defined as areas of high signal surfacing on the superior and/or inferior surface or displaced meniscal tears.
	Chondral lesion	Simply defined as an observed chondral defect.
	Anterior tibial subluxation	Measured relative to a posterior femoral condylar reference line on sagittal proton density images.
Osaki et al.	Cartilage degeneration	T1p values measured relative to certain areas of interest in the medial femoral cartilage.
	Anterior displacement difference (ADD)	Measured by anterior drawer stress radiography.
Yoon et al.	PCL angle	T2‐weighted fat‐suppressed magnetic resonance image, which was defined as the angle between lines drawn through the central portions of the tibial and femoral insertions of the posterior cruciate ligament.
Liu et al.	Anterior subluxation of lateral compartment	The slice showing the most medial edge of fibula at the tibiofibular joint was selected. At first, a best‐fit circle tangent to the posterior femoral condyle border was depicted. Along the posterior margin of the circle, a line (line a) was drawn vertically to the tibial plateau (line b), and another parallel line (dotted line c) was drawn across the posterior aspect of tibia. The distance (d, from line a to dotted line c) was the amount of anterior tibial subluxation of the lateral compartment.
	Anterior tibial subluxation of medial compartment	The first slice with medial gastrocnemius tendon on the femur was chosen. At first, a best‐fit circle tangent to the posterior femoral condyle border was depicted. Along the posterior margin of the circle, a line (line a) was drawn vertically to the tibial plateau (line b), and another parallel line (dotted line c) was drawn across the posterior aspect of tibia. The distance (d, from line a to dotted line c) was the amount of anterior tibial subluxation of the medial compartment.
	Lateral posterior tibial slope	The central sagittal slice was first confirmed, containing intercondylar eminence, anterior and posterior tibial cortices appearing as a concave shape, and tibial attachment of posterior cruciate ligament. A circle tangent to the proximal, anterior, and posterior tibial borders and a second circle centred on the perimeter of the first circle was drawn, followed by connecting the centres (red points) of two circles to define the tibial longitudinal axis (line a). Then, the angle between the line vertical to the tibial longitudinal axis (line b) and the tangent to the lateral tibial plateau (line c) was defined as the lateral posterior tibial slope and measured.
	Medial posterior tibial slope	The central sagittal slice was first confirmed, containing intercondylar eminence, anterior and posterior tibial cortices appearing as a concave shape, and tibial attachment of posterior cruciate ligament. A circle tangent to the proximal, anterior, and posterior tibial borders and a second circle centred on the perimeter of the first circle was drawn, followed by connecting the centres (red points) of two circles to define the tibial longitudinal axis (line a). Then, the angle between the line vertical to the tibial longitudinal axis (line b) and the tangent to the medial tibial plateau (line c) was defined as the medial posterior tibial slope and measured.
Ye et al.	ATS	Measured the ATSMC and ATSLC on the sagittal T1WI slices that showed the femoral insertion of the medial gastrocnemius tendon and the medial aspect of the fibula at the tibiofibular joint, respectively (Figure 3). The best‐fit circle was drawn over the subchondral line of the posterior femoral condyle. Two lines were drawn perpendicular to the tangent of the tibial plateau and tangential to (1) the posterior margin of the best‐fit circle and (2) the posterior aspect of the tibia. The distance between these two parallel lines was determined as the ATSMC or ATSLC.
	Posterior tibial slope	In brief, the centres of the cranial and caudal circles were connected to determine the tibial longitudinal axis on the central sagittal slice; the medial tibial slope and lateral tibial slope (LTS) were measured as the angles between the perpendicular line of the tibial longitudinal axis and the tangential line of the medial and lateral tibial plateaus on their midsagittal slices, respectively.
Wang et al.	Inter‐condylar notch width (ICW) and femoral condylar width (FCW)	Patients received 3.0 T MRI with a scanning thickness of 3 mm. T2 coronary view with fat suppression was scanned anterior to posterior. On the first image where the intercondylar notch appeared completely, the femoral ICW and FCW were measured along a line parallel to the most inferior femoral condylar line.
Vlychou et al.	Cartilage lesion	MR grading of the cartilage lesions was based on the International Cartilage Repair Society classification for traumatic cartilage lesions and compared directly with arthroscopic findings as defined by the Outerbridge classification. Normal cartilage was scored as grade 0 and the threshold of 50% was used for MR evaluation as follows: grade I for superficial lesions such as fissures and cracks, grade II for lesions extending down to <50% of cartilage depth, grade III for defects extending down >50% of cartilage depth and grade IV for full‐thickness lesions with subchondral bone exposure.
Xu et al.	Anterior tibial displacement	Refers to increased distance from the posterior cortex of the lateral tibial plateau to the posterior cortex of the femur.
van Meer et al.	Cartilage lesion	Cartilage lesions were scored as follows: each articular cartilage region was graded for size of any cartilage loss (as a percentage of surface area as related to the size of each region surface) and for percentage of loss in this subregion that is full‐thickness loss. These scores were categorised from 0 to 3.
Oronowicz et al.	PCL–PCA	For the measurement of the PCL angle, the PCL–PCA was assessed on sagittal T2‐weighted images with an accuracy of 0.1°. It was calculated as the angle between a line drawn on the central portion of the PCL at its most vertical part and a second line defined by the posterior diaphyseal cortex of the femur.
Gong et al.	PCL‐index (PCLI)	The most complete ligament contour was found on sagittal T2‐weighted imaging (T2WI) scans. First, a line between the tibial and femoral attachments of the PCL was drawn(X)—from the lower edge of the PCL tibial attachment to the posterior edge of the femoral attachment. Then, a vertical line perpendicular to this line was drawn through the highest point of the PCL (Y)—lower margin of PCL profile. The ratio of the two lines was the PCLI (X/Y).
	Lateral femoral notch sign	For the LFNS measurements, the deepest supracondylar notch was found on sagittal MRI scans. First, a tangent line was drawn along the cortex of the lateral femoral condyle. Then, a vertical line perpendicular to the tangent line was drawn through the highest point of the notch. The length of the vertical line was equal to the depth (mm) of the notch.
Zhang et al.	Global anterior tibial subluxation	represents the overall anteroposterior tibiofemoral position, calculated by averaging the values of LATS and MATS.
Mishima et al.	Anterior tibial subluxation	Tracing of the contour of the unaffected knee. (B) Tracing of the contour of the unaffected knee is superimposed on the image of the affected knee, and the contours of the bilateral femurs are matched. The tibia of the affected knee shows anterior subluxation. (C) The tracing of the contour of the unaffected knee is then moved along the tibial joint line relative to the image of the affected knee, so that the tracing of the contours of the tibiae match. The distance moved indicates the extent of subluxation.

Abbreviations: ALL, anterior‐lateral ligament; LATS, lateral anterior tIbial subluxation; MATS, medial anterior tibial subluxation; MRI, magnetic resonance imaging; PCL, posterior cruciate ligament.

### MRI findings—meniscus

#### Medial meniscus

Nine studies reported findings related to medial meniscus (MM) injuries [8, 25, 29, 37, 49, 52, 53, 54, 60]. Five studies reported higher rates of MM tears in the chronic ACL group compared to the acute comparators (Dimond et al. [79% vs. 23%]; [8] McDonald et al. [67.5% vs. 49%]; [29] Osaki et al. [71% vs. 32%]; [37] Liu et al. [25] [34.6% vs. 10.5%]); one reported similar rate (Xu et al. [52] [28.5% vs. 24%], *p* = 0.282). Ye et al. reported that posterior horn MM tears independently predicted knee laxity (odds ratio [OR], 2.62; 95% confidence interval [CI], 1.35–5.08; *p* = 0.004) [53]. Cohort studies reporting MM tear rates included Zeng et al. (61.1%) [58], Vlychou et al. (39.5%) [49] and Yoo et al. [54] (55%–84% at 6 months after initial measurement, during conservative management of ACL injuries).

Meta‐analysis found that at a mean of 26.8 days post‐injury, the acute ACL group had a 36.0% prevalence of MM tears. Meanwhile, at a mean of 816.5 days post‐injury, the chronic ACL group reported a statistically greater prevalence of 50.3% MM tears (OR: 2.99, 1.45–6.13, *n* = 769, *I* ^2^ = 74%; Table 2, Figure 2). Statistical modelling using the akaike information criterion demonstrated that a linear model provided the best fit to describe the relationship between time and the prevalence of MM tears (*R* ^2^ = 0.20, Figure 3).

**Figure 2:**
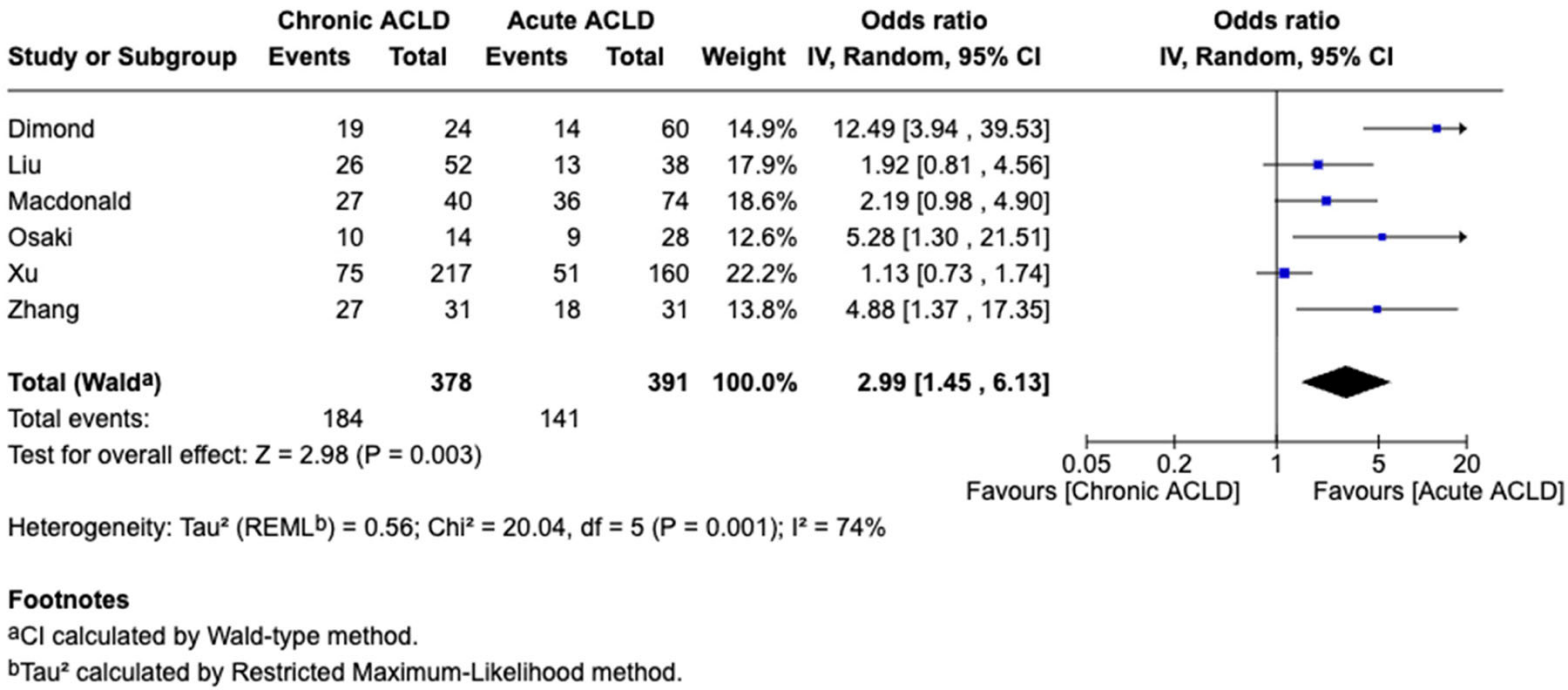
Higher odds ratio of medial meniscus tears in chronic anterior cruciate ligament deficiency (ACLD) patients compared to acute ACLD patients. CI, confidence interval.

**Figure 3:**
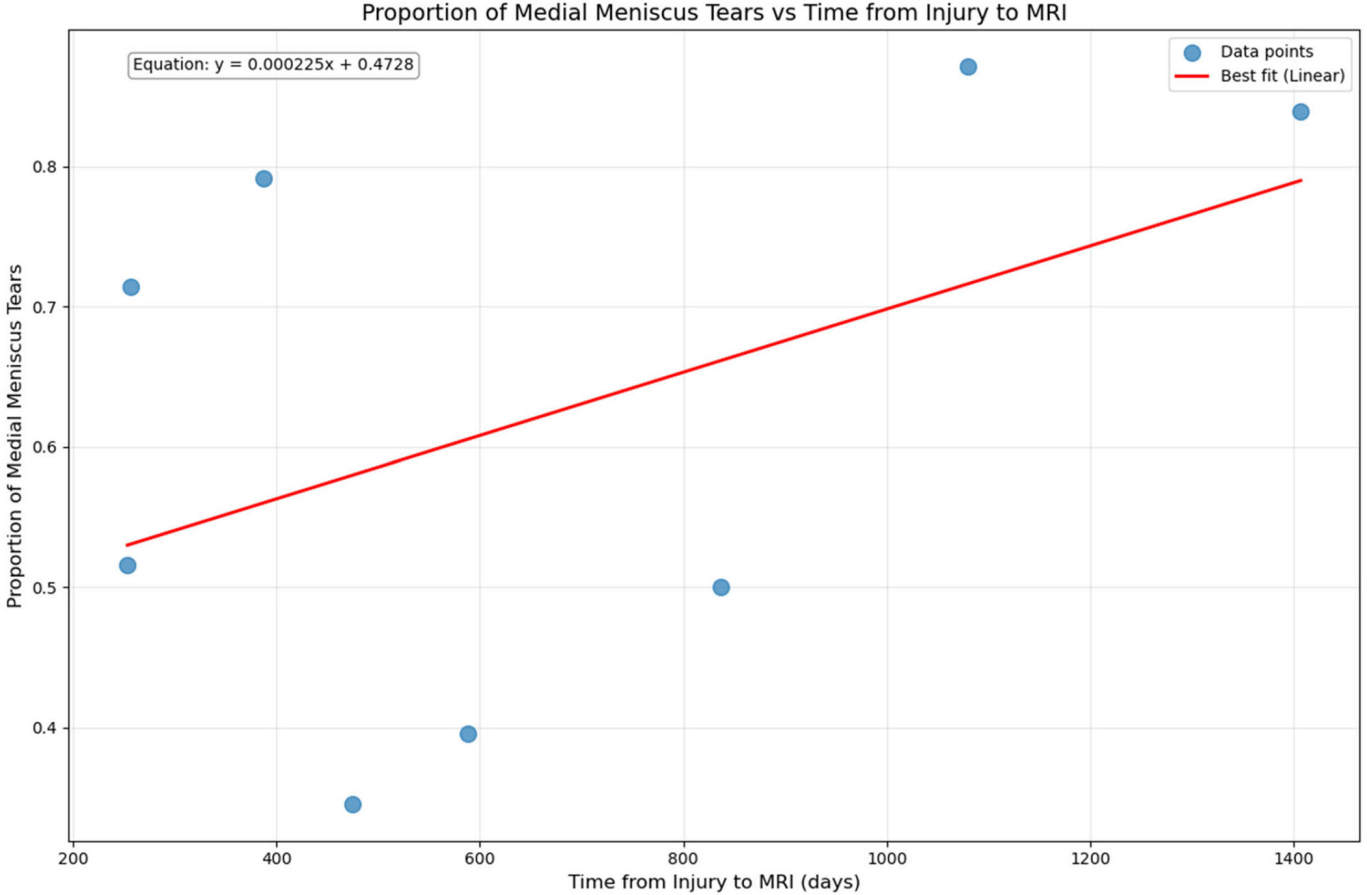
Logarithmic relationship between the proportion of medial meniscus tears and time since injury (*R* ^2^ = 0.20).

#### Lateral meniscus

Nine studies reported findings related to lateral meniscus (LM) injuries [8, 25, 29, 34, 37, 49, 52, 53, 60]. Four studies reported no difference between chronic and acute patients (Dimond et al. [21% vs. 27%]; [8] McDonald et al. [45% vs. 43%]; [29] Osaki et al. [43% vs. 43%]; [37] Xu et al. [21.6% vs. 27.5%]; [52] Liu et al. [13.5% vs. 31.5%] [25]. Ye et al. [53] reported that posterior horn LM tears were an independent risk factor for knee laxity (OR, 2.39; 95% CI, 1.20–4.78; *p* = 0.014). Vlychou et al. [49] reported a LM tear rate of 14/43 (32.5%). Nonaka et al. [34] reported a LM tear rate of 20/29 (69%) in patients with ramp lesions and 15/21 (71%) in patients without ramp lesions.

Pooled analysis found that at a mean of 816.5 days post‐injury, the chronic ACL group reported a prevalence of 29.4% of LM tears, while at a mean of 26.8 days post‐injury, the acute ACL group had a 37.9% prevalence of LM tears. Pooled analysis of meniscus tears (Figure 4, Supporting Information S1: Table S3) showed no difference in lateral meniscus injuries between acute and chronic ACLD groups.

**Figure 4:**
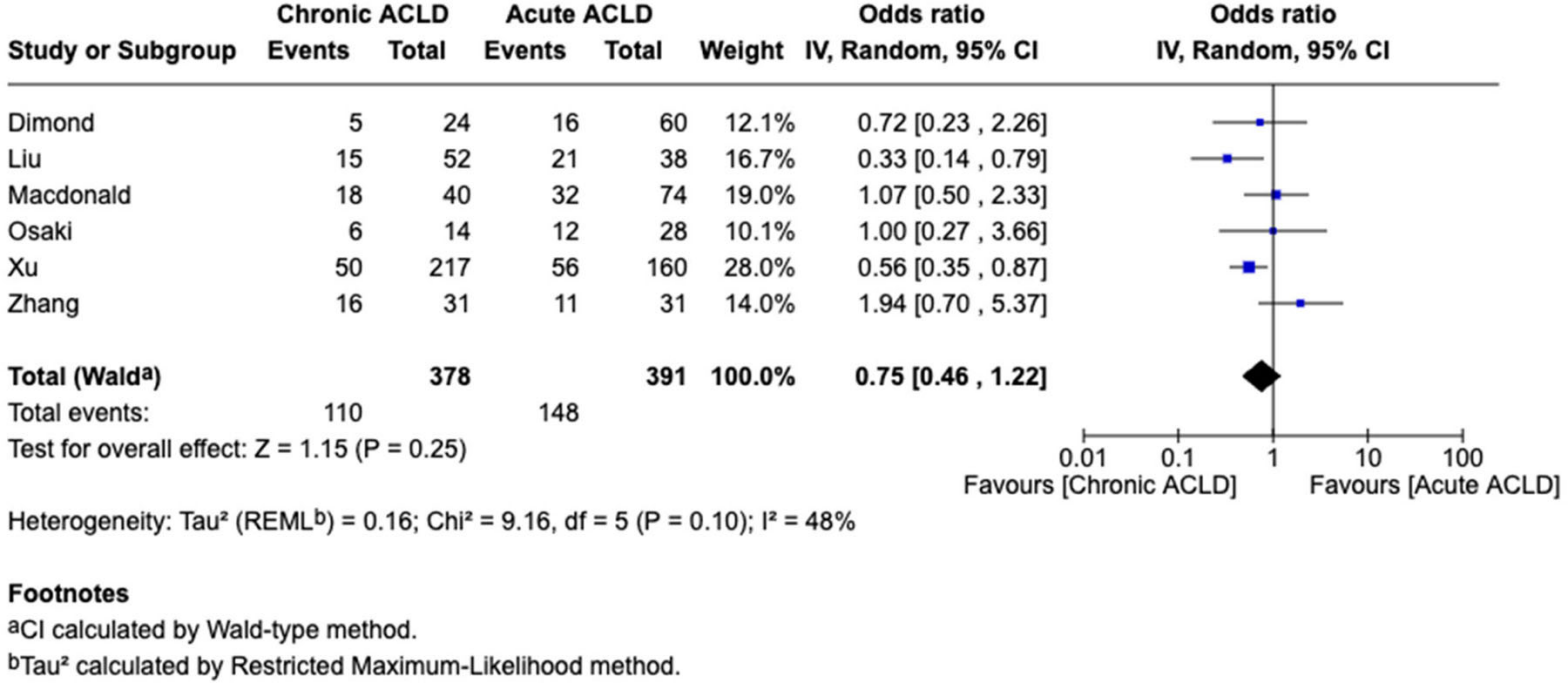
No difference in lateral meniscus tear rates between acute and chronic anterior cruciate ligament deficiency (ACLD) groups.

### MRI findings—cartilage lesions

Five studies reported findings related to cartilage lesions on MRI [6, 8, 29, 37, 58]. Osaki compared cartilage degeneration in chronic ACLD knees to healthy controls, and acute cases. Chronic patients had higher T1ρ values, indicating degeneration, in the anterior and middle medial femoral condyle than all other groups (*p* < 0.05) [37]. Conversely, Crook et al. [6] reported that there were elevated cartilage strains in ACL‐deficient knees compared to the healthy knees (4% vs. 3%, *p* = 0.01). Zeng et al. reported that the anterior MFC had more severe cartilage lesions compared to the posterior and middle sections (*p* = 0.001) [58]. The posterior medial and lateral tibial plateaus had more severe lesions than the other areas (anterior and middle, *p* = 0.01) McDonald et al. [29] reported a 15% (11/74) rate of MFC defects in acute versus chronic ACLD patients (67.5% [27/40], *p* = 0.001). Dimond et al. reported five cases of chronic ACLD knees that had ‘Focal erosion and/or diffuse thinning, subchondral involvement, on the articular surface adjacent to medial meniscus’, compared to none in the acute ACL cohort [8]. Please refer to Supporting Information S1: Table S2 for all findings related to cartilage lesions. Heterogeneity of cartilage lesion outcome reporting precluded meta‐ or pooled analysis.

### MRI findings—tibial subluxation and translation

Seven studies reported findings on tibial subluxation and translation [25, 29, 30, 37, 52, 53, 60]. With respect to anterior medial tibial subluxation (AMTS) in chronic versus acute ACL‐injured knees, McDonald et al. [29] (0.9 mm [2.4] vs. 0.1 mm [2.5] vs. intact controls: −0.9 mm [2.1]), while Liu et al. [25] reported (+4.5 mm [1.0] vs. +2.3 mm [0.5]). With respect to anterior lateral tibial subluxation (ALTS) in chronic versus acute ACL‐injured knees, McDonald et al. [29] reported (3.6 mm [3.9] vs. 2.8 mm [3.2] vs. intact controls: −0.8 mm [2.7], *p* < 0.001), while Liu et al. [25] reported (+7.4 mm [0.5] vs. +6.2 mm [1.0]). Ye et al. [53] reported that patients with high‐grade knee laxity had higher AMTS (+2.3 mm, ±2.8 mm) compared to the low‐grade chronic ACLD knees (+0.6 mm, ±2.9 mm), and higher ALTS (+6.2 mm, ±3.7 mm) versus (+3.5 mm, ±3.3 mm) (*p* < 0.001).

With respect to global anterior tibial subluxation (GATS), Osaki et al. [37] reported (Group C: +6.5 mm [4.7], Group B: +5.9 mm [3.1], Acute: +5.8 mm [3.3] compared to control). Xu et al. [52] reported the threshold for anterior displacement was met in 38/160 (23.8%) acute ACLD patients and 58/217 (26.7%) chronic patients (*p* = 0.349). Mishima et al. [30] reported +2.1 mm (1.3) GATS compared to healthy contralateral knees, and a positive correlation between GATS and time since injury (*r* = 0.332, *p* < 0.0013). Zhang et al. [60] reported no difference in AMTS, ALTS, or GATS in pre‐operative MRIs of acute and chronic ACL‐injured knees.

Pooled analysis found increased anterior medial tibial translation (Figure 5a), but not anterior lateral tibial translation (Figure 5b) in the chronic ACLD group. Heterogeneity prevented time‐based plotting.

**Figure 5:**
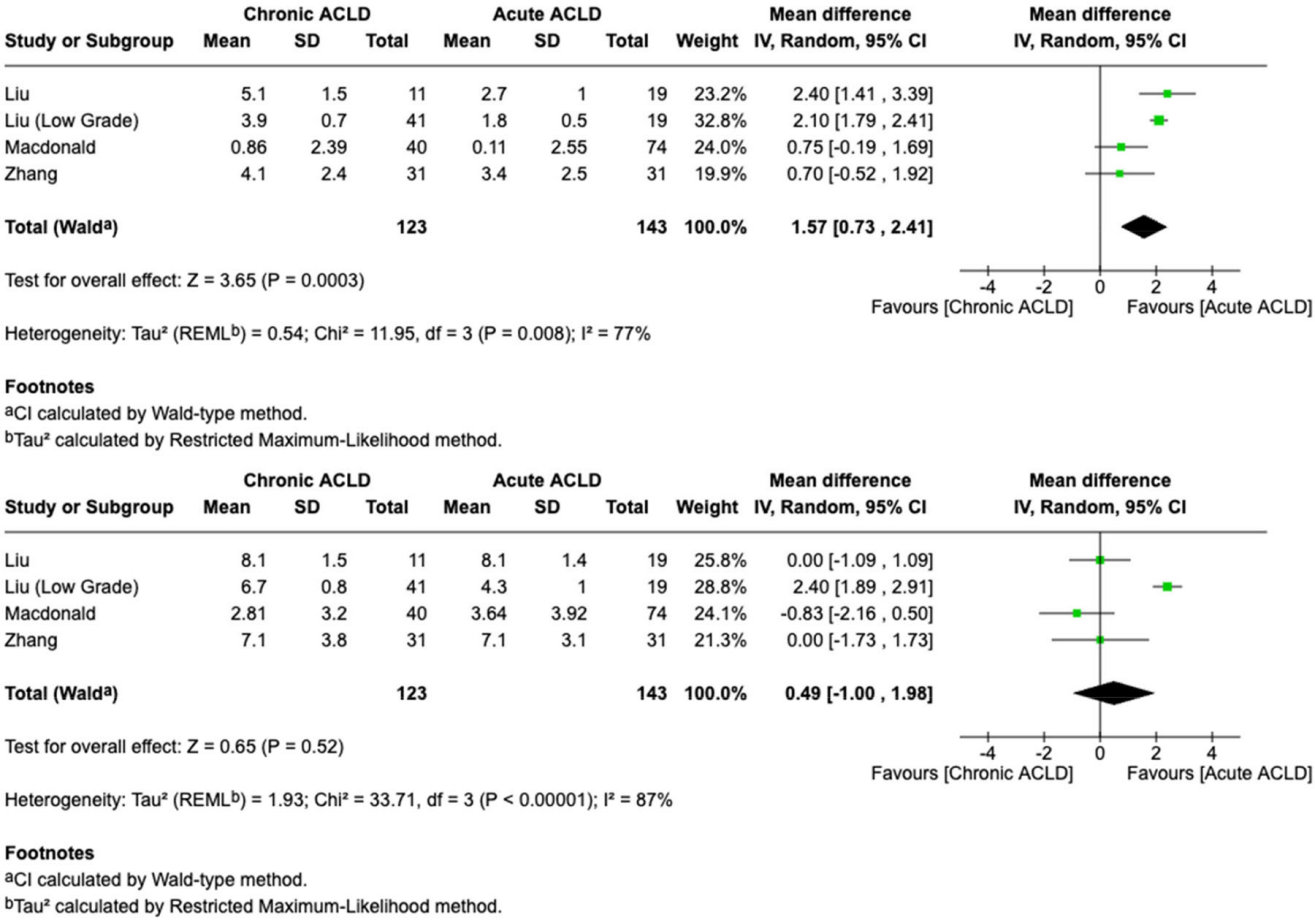
Increased anterior medial tibial subluxation (a) but not lateral (b) subluxation in chronic anterior cruciate ligament deficiency (ACLD) knees. CI, confidence interval.

### MRI findings—PCL angle

Five studies reported on PCL morphology [8, 13, 36, 56, 57]. Dimond et al. [8] found increased mean PCL bowing ratio in chronic ACLD knees (0.47 [0.11] versus 0.37 [0.12], *p* = 0.013). Yoon et al. [56] found lower PCL angles over time (chronic: 98.7 ± 18.2°, intermediate: 110.4 ± 15.4°, subacute: 116.2 ± 14.1°, acute: 125.1 ± 15.1°). Yoon et al. [57] reported that the PCL angle decreased with chronicity of injury (Spearman coefficient, −0.567; *p* < 0.001). Oronowicz et al. [36] reported that the median PCL–PCA of the entire population was 16.2° (range: 4.8–49.6°). The median PCL‐PCA for the chronic group was 10.7° (7.4–17.2°); significantly lower (−7.7°) in chronic injuries compared to acute injuries (*p* < 0.01). Gong et al. [13] reported a decrease in the PCL Index (chronic: 4.8 ± 1.4, subacute: 5.1 ± 1.6, acute: 5.3 ± 1.7; *p* < 0.004), likely due to tibial translation.

### MRI findings—ACL appearance findings

Dimond et al. [8] reported acute ACL tears had a higher percentage of diffuse increased signal intensity (*n* = 35, 58% of knees) and focal intensity (*n* = 25, 42% of knees) compared to those in the chronic group (*n* = 4, 15% and *n* = 3, 11%) of chronic ACLD knees (Figure 6). Eight chronic knees (30% of knees) had a band of low signal intensity, and 12 showed a fragmented ACL on MRI, while no acute knees had either. Lastly, a similar number of acute and chronic knees (12%–11%) showed a hyperintense signal adjacent to the torn ACL.

**Figure 6:**
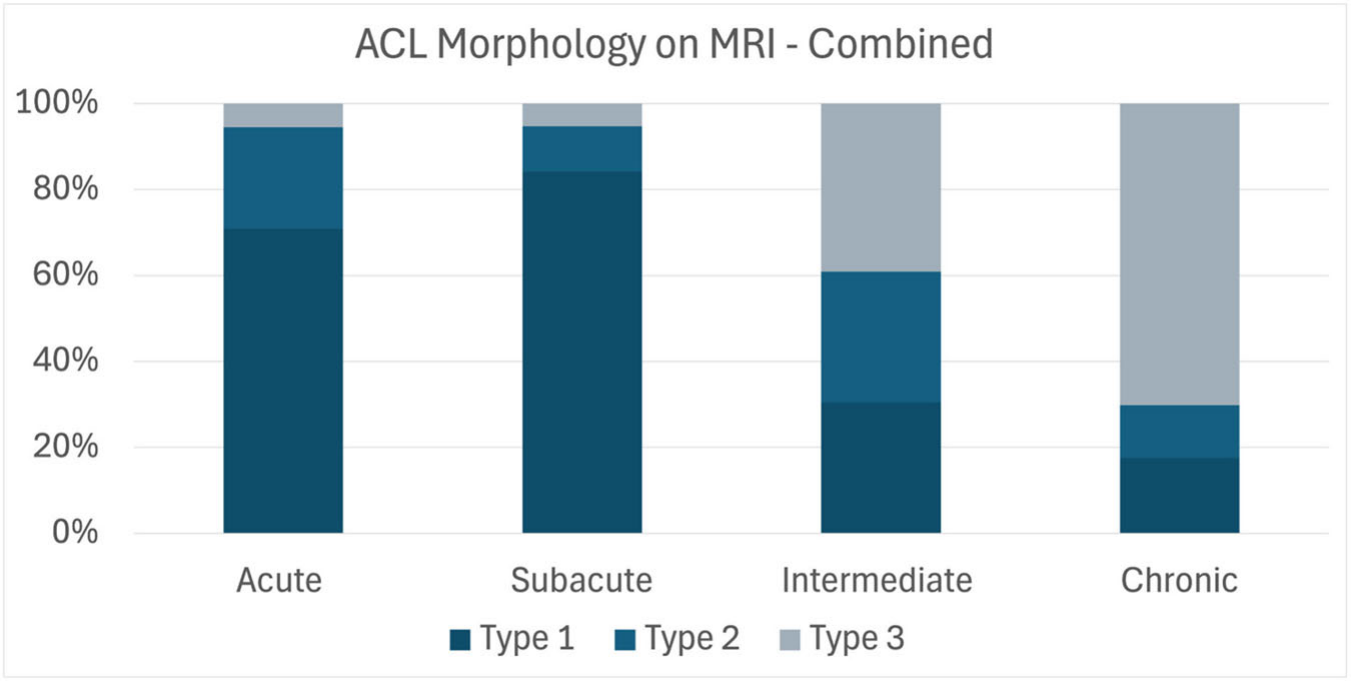
Combined anterior cruciate ligament (ACL) morphology ratios across Yoon et al. [56] and Dimond et al. [8]. MRI, magnetic resonance imaging.

Vahey et al. [47] found that of the 30 chronic tears documented at arthroscopy, 20 were correctly diagnosed on MRI. 11/20 (55%) demonstrated visible fragments without associated oedema. These fragments usually had an abnormal course and scarred down to the posterior cruciate ligament. 4/20 (20%) demonstrated nonvisualization of the ligament without associated oedema. 5/20 (25%) demonstrated an intact band that bridged the expected origin and insertion of the ACL but had a discrete focal angulation.

Yoon et al. [56] stratified ACL appearance on MRI into three types of morphologies: Type 1 (increased signal intensity and an oedematous mass‐like shape), Type 2 (increased signal intensity with a fragmented shape), Type 3 (low signal intensity and a band‐like fragmented shape or nonvisualization of the anterior cruciate ligament). For the exact numbers and percentage of each of the types within the chronicity groups, please refer to Supporting Information S1: Table S2. The ratio of type 3 morphologies increased as the time from injury increased (*p* < 0.001) (Figure 6).

### Other findings on MRI

Ates et al. reported the healing of the anterolateral ligament (ALL) in acute and chronic ACLD knees at the time of injury and pre‐operatively [2], using Muramatsu grading to quantify the ALL healing outcome (Grade A: ALL is continuous and clearly defined low signal band, Grade B: ALL bending, thinning or isosignal changes, Grade C: no clear and continuous signal band). The chronic group had more Group C ALLs. Wang et al. [50] reported on MRI decreased inter‐condylar notch width (ICW) in chronic patients, implying the development of notch stenosis (Supporting Information S1: Table S2). Within studies that reported posterior tibial slope by chronicity, the authors did not observe a change given slope is constant and this has been tracked as a form of internal validation (Supporting Information S1: Table S2). Five studies [8, 31, 34, 56, 57] reported on features such as bone bruising/oedema and intra‐articular knee effusion. All studies reported that bone bruising and effusion were prevalent in the acute group and decreased in the chronic group.

## DISCUSSION

This systematic review is consistent with the fact that medial compartment changes tend to develop over time in the chronic ACLD knee, in agreement with previous studies. Specifically, chronicity was associated with significantly increased rates of medial meniscus tears in a time‐dependent manner, and an increase in anterior medial tibial translation, while increased cartilage strain and lesions were observed on the medial femoral condyle, and medial tibial plateau. Expected findings were seen in terms of the PCL, with increased buckling, and the ACL, with fragmentation and disappearance of the remnant, and bone bruising and joint effusion, with both resolving over time.

In comparison to acute ACLD knees, chronic ACLD knees show higher rates of medial sided changes, particularly with regards to the meniscus, cartilage damage and anterior translation. Pooled analysis of matched studies showed a medial meniscus tear odds ratio of 2.99 in the chronic group (50.3% vs. 36.0%, *p* < 0.001) at 816.5 days post‐injury (Figure 2). Temporal modelling showed a linear relationship over time (*R* ^2^ = 0.20) (Figure 3). Interestingly, the chronic and acute ACLD groups showed no difference in lateral meniscus tears (29.4% vs. 37.9%, *p* = 0.25) and no difference in anterior lateral subluxation (*p* = 0.52) (Figures 4 and 5b).

The finding of accelerated chondral degeneration has been reported in large database research [12]. Cadaveric work has shown that chronic ACL deficiency leads to increased loading and shear forces in response to valgus and internal rotation torque in the medial tibiofemoral cartilage, which was seen in this study, and the lateral meniscus, which was not observed [28]. Laboratory studies confirmed that hopping increased medial tibiofemoral cartilage strain, particularly with greater chronicity from injury [43]. In vivo work shows that the contact points of the medial tibiofemoral joint shift laterally and posteriorly during lunges and squats, which explains some of the observed wear patterns in the medial compartment [17, 23].

Longitudinal imaging and clinical studies confirm that medial meniscal extrusion and degeneration progress after ACL injury and are associated with subsequent cartilage degeneration and osteoarthritis, indicating a bidirectional relationship: meniscal changes are initially a result of ACL deficiency but then act as a driver of further joint degeneration [21, 51]. Longitudinal research suggests that knee arthritis progression differs by compartment, with cartilage loss initiating changes centrally and meniscal pathology leading posteriorly [22]. Early meniscal preservation or repair after ACL injury may mitigate subsequent joint degeneration and progression of osteoarthritis, but the protective effect is incomplete and depends on timing, meniscal status, and surgical technique [1, 4, 27]. Secondary tears often present as complex tears of the medial meniscus and result in high rates of partial meniscectomy [14]. Meanwhile, consensus states early ACL reconstruction (within 5–12 weeks of injury) reduces the risk of secondary meniscal tears and subsequent cartilage damage compared to delayed or nonoperative management [39].

Although this study's definition of chronic ACLD knee was >6 months post‐injury [11], a review of the literature on studies with 3–6‐month post‐injury subgroups showed that these ‘chronic’ ACLD changes may begin earlier than the arbitrary time‐point of 6 months. Anatomic changes that begin to develop in this ‘subacute’ phase include increased cartilage relaxation time, decreased cartilage thickness, worsened by ipsicompartmental meniscus tears [5, 44]. However, many knees in the ‘chronic’ group of this study did not demonstrate anatomical changes. Therefore, the approach to an ACL‐deficient knee classified by an arbitrary temporal relationship to the injury may be a less informative approach than categorising ACL‐injured knees by those with and without secondary anatomical changes.

Clinical cohort studies further confirm that delaying ACL reconstruction beyond 12 weeks is associated with a higher prevalence of medial meniscal tears and early chondral pathology post‐operatively. Zheng et al. reports time from injury to reconstruction >12 weeks was a strong independent predictor of anterior lateral tibial translation (OR, 17.1) [20, 61]. A meta‐analysis found that earlier ACL reconstruction (<6 or 12 weeks) was predictive against medial meniscus and chondral injuries in paediatric patients [19], as did a large cohort study with >5 months to reconstruction [15]. Other studies suggested that the chronicity of ACL injury to reconstruction predicts the post‐surgical frequency of medial/lateral meniscal injury, severity and presence of chondral lesions, graft failure rates [45], less sports participation [32, 46] and medial/global tibial subluxation [60] in a time‐dependent manner.

Other findings, including increased PCL buckling in chronic knees, fragmentation or resorption of the ACL remnant, ALL attrition, reduced bone bruising, and reduced knee effusions were in alignment with expectations. Notably, the appearance of the chronically torn ACL does have a consistent appearance on MRI, and in some cases can be misdiagnosed as a partial tear or intact ligament. This paper also highlighted a study which utilised logistic regression models to accurately predict chronicity in ACL deficiency through MRI imaging findings [57]. Yoon et al. found that certain MRI findings such as ACL morphology types, bone bruising, PCL angle, and effusion may effectively predict chronicity. Other literature suggests that PCL buckling may be improved partially by ACL reconstruction, although the clinical benefit remains unknown [55].

### Limitations

The primary limitation of this systematic review and meta‐analysis is heterogeneity of data reporting in included studies, which limits the reliability of findings and the ability to present accurate summary statistics. Pooled analysis of secondary data is accurate only to the information available, and important secondary factors predicting anatomical knee changes other than ‘chronicity’ are not evaluated (e.g., coronal plane alignment, BMI, activity level). Additionally, due to inherent bias in studies, some statistical ranges varied greatly (e.g., anterior medial tibial translation ranged from +0.9 to +5.1 in chronic ACLD knees), and some studies dichotomised continuous outcomes (e.g., anterior translation reported as a binary variable in Xu et al.). The authors opted to mitigate this bias, by performing meta‐analysis with only matched‐control cohort studies, to improve group similarity and measurement internal validity. A secondary limitation of the study is that the articles included range from 1991 to 2024, and thus reporting of MRI findings and MRI technology can be inconsistent across decades, adding heterogeneity to the study. Furthermore, the inclusion of MRIs in chronic ACL‐deficient patients planning to undergo delayed ACL reconstruction implies the inclusion of bias towards a symptomatic cohort, causing over‐representation of meniscocartilaginous injuries. Unfortunately, few studies routinely conducted MRIs in non‐surgical asymptomatic patients. Another inherent limitation with observation research is that MRI may not be sensitive enough to detect subtle meniscus and cartilage abnormalities in the early stages of ACL deficiency [40]. Another limitation is the accuracy of MRI in detecting intra‐articular injuries. Although many studies report 95%+ specificity and sensitivity in detecting meniscal tears, studies did not confirm MRI findings with arthroscopy. This may lead to under‐reporting of meniscochondral injuries in both groups. Given that analysis was performed on matched cohort studies, this would likely not dramatically change findings but may suggest that the pooled rates of injury are under‐reported.

## CONCLUSION

This systematic review of MRI‐identified anatomical changes in the chronic (>6 months) ACL‐deficient knee found a time‐dependent higher rate of medial meniscus injuries, medial compartment cartilage lesions, AMTS, ACL attenuation and PCL verticality. Some studies suggest that these changes can be seen even before 6 months from injury while not every chronic knee has anatomical abnormalities. The exact clinical significance of anatomical changes is not fully understood, but the present study lends credence to the notion that a more accurate anatomical categorisation of the ACL‐deficient knee is those with and without structural changes—a factor influenced by time. Clinically, these changes can help guide clinicians in evaluating the chronicity of an ACL deficiency with related changes over time. Furthermore, clinicians can council patients on what to expect to experience and see over the course of ACL deficiency.

## AUTHOR CONTRIBUTIONS

Abel Davtyan was responsible for study ideation, literature search, screening, data extraction, statistical analysis, manuscripts writing, edition and revisions. Ajay Shah was responsible for data extraction, analysis, manuscript writing, edition and revision. Quang Le, Ananya Pathak and Veronika Kolarska were responsible for screening, manuscript writing, and revisions. All other authors were involved in edition, revision and supervision.

## CONFLICT OF INTEREST STATEMENT

The authors declare no conflicts of interest.

## ETHICS STATEMENT

The authors have nothing to report.

## Supporting information

Supplementary File.

## Data Availability

All data used for this review are available in several databases and are cited in this journal submission.
