# Supplementary material for: Magnetic resonance imaging findings in chronic anterior cruciate ligament‐deficient knees: A systematic review of meniscal, cartilage, tibial translation and ligamentous changes
Source: J Exp Orthop. 2026 Mar 24;13(1):e70696. doi: 10.1002/jeo2.70696 (PMC13679891; doi:10.1002/jeo2.70696)
Supplement: Supplementary file 1 [file JEO2-13-e70696-s001.docx]

**Supplementary Table 1. Methodological quality assessment of included studies using the MINORS criteria**

| **Study Name** | **MINORS Score** |
| --- | --- |
| A comparison of MRI findings in patients with acute and chronic ACL tears. | 19 |
| Acute and chronic tears of the anterior cruciate ligament: differential features at MR imaging. | 19 |
| Chronic ACLD Knees with Early Developmental Cartilage Lesions Exhibited Increased Posterior Tibial Translation during Level Walking. | 20 |
| Effect of walking on in vivo tibiofemoral cartilage strain in ACL-deficient versus intact knees. | 20 |
| Evaluation of anterolateral ligament healing in patients with anterior cruciate ligament injury without anterior cruciate ligament reconstruction and its relationship with clinical examination tests. | 20 |
| Passive Anterior Tibial Subluxation in the Setting of Anterior Cruciate Ligament Injuries: A Comparative Analysis of Ligament-Deficient States. | 19 |
| Characterization of Biochemical Cartilage Change After Anterior Cruciate Ligament Injury Using T1rho Mapping Magnetic Resonance Imaging. | 16 |
| Correlation of magnetic resonance imaging findings with the chronicity of an anterior cruciate ligament tear | 19 |
| Osseous lesions associated with anterior cruciate ligament injuries. Assessment by magnetic resonance imaging at various periods after injuries. | 18 |
| Prediction of chronicity of anterior cruciate ligament tear using MRI findings | 19 |
| Anterior Tibial Subluxation of Lateral Compartment Is Associated With High-Grade Rotatory Instability for Acute But Not Chronic Anterior Cruciate Ligament Injuries: An Magnetic Resonance Imaging Case-Control Study. | 20 |
| Association Between Anterior Tibial Subluxation of Lateral Compartment and High-Grade Knee Laxity in Patients With Anterior Cruciate Ligament Deficiency. | 23 |
| Association between time from anterior cruciate ligament injury to reconstruction and morphological changes of the intercondylar notch using MRI and arthroscopy. | 22 |
| Chronic ACL-injured patients show increased medial and global anterior tibial subluxation measured on 1-year postoperative MR images after primary single-bundle ACL reconstruction. | 22 |
| Chronic anterior cruciate ligament tears and associated meniscal and traumatic cartilage lesions: evaluation with morphological sequences at 3.0 T. | 22 |
| Comparison of magnetic resonance imaging for patients with acute and chronic anterior cruciate ligament tears. | 19 |
| Degenerative Changes in the Knee 2 Years After Anterior Cruciate Ligament Rupture and Related Risk Factors: A Prospective Observational Follow-up Study | 22 |
| Increasing incidence of medial meniscal tears in nonoperatively treated anterior cruciate ligament insufficiency patients documented by serial magnetic resonance imaging studies. | 19 |
| Posterior Displacement of Meniscal Ramp Lesion Is Observed on Flexed Knee Magnetic Resonance Imaging of Anterior Cruciate Ligament-Deficient Knees | 21 |
| The Posterior Cruciate Ligament Posterior Cortex Angle (PCL-PCA), an early sign of sagittal decompensation in the ACL-deficient knee | 20 |
| The posterior cruciate ligament index as a reliable indirect sign of anterior cruciate ligament rupture is associated with the course of knee joint injury. | 20 |

MINORS items assessed: (1) clearly stated aim; (2) inclusion of consecutive patients; (3) inclusion of consecutive patients; (3) prospective collection of data; (4) endpoints appropriate to the aim of the study; (5) unbiased assessment of the study endpoint; (6) follow-up period appropriate to the aim of the study; (7) loss to follow-up less than 5%; (8) prospective calculation of the study size. Each item was scored as 0 (not reported), 1 (reported but inadequate), or 2 (reported and adequate). Maximum score for non-comparative studies is 16.

**Supplementary Table 2. MRI Findings (Full)**

| **Author** | **MRI outcomes** | **Cohort Matched (Y/N)** | **Comparative (Y/N)** | **Comparator Group** | **Time from Injury to MRI (Mean)** | **ACL Appearance** | **Meniscus Lesions - Medial, Lateral** | **Cartilage Lesions - Patellofemoral, MFC, LFC, MTP, LTP** | **PCL Appearance** | **Other Ligament Injury (ALL, PCL, LCL, MCL)** | **Tibial Subluxation and Internal Rotation** | **Posterior Tibial Slope** | **Other Findings** |
| --- | --- | --- | --- | --- | --- | --- | --- | --- | --- | --- | --- | --- | --- |
| Dimond et al | ACL appearance, Meniscal tears, Cartilage injury, PCL bowing ratio | N | Y | Acute ACL tear | Acute: 2.5 weeks (1 day - 5.7 weeks)  Chronic: 55.4 weeks (26.4 weeks - 9.9 years) | **Acute**: 58% had diffuse increased signal, 42% had focal increased signal. **Chronic**: 15% had diffuse increased signal, 11% had focal increased signal. 44% showed fragmented ligament, 30% showed a band of low-signal intensity. | **Acute**: 8 isolated medial tears + 6 combined medial & lateral tears (14/30), 10 isolated lateral meniscus tears + 6 combined medial & lateral tears (16/30)  **Chronic**: 16 isolated medial tears + 3 combined medial & lateral tears (19/24), 2 isolated lateral meniscus tears + 3 combined medial & lateral tears (5/24) | MFC: Acute: 0, Chronic: n=5 cases, Focal erosion and/or diffuse thinning, subchondral involvement, articular surface adjacent to medial meniscus | PCL bow ratio: Chronic: 0.47 (0.11). Acute: 0.37(0.12). (p = 0.013). | Acute: 13 MCL injuries, Chronic:0 MCL injury | NR | NR | NR |
| Vahey et al | ACL appearance | N | Y | Acute ACL tear, Healthy control | Acute: 17 days (0-42 days),  Chronic: 5.5 years (6 months - 21 years). | Of the 30 chronic tears documented at arthroscopy, 20 were correctly categorized at MR.11/20 demonstrated visible fragments without associated edema. The residual fragments usually had an abnormal course as they typically scarred down to the posterior cruciate ligament. 4/20 demonstrated nonvisualization of the ligament without associated edema. 5/20 demonstrated an intact band that bridged the expected origin and insertion of the ACL but had a discrete focal angulation. | NR | NR | NR | NR | NR | NR | Fibrous scarring within the intercondylar notch is a key finding in chronic tears |
| Zeng et al | ACL appearance, Cartilage defects, Subchondral bone attrition | N | Y | Healthy controls | Cartilage lesions group: 12.7±1.4 months,  No cartilage lesions group: 13.6±1.5 months | No details on intraligamentous signal intensity, ligament length, or bowing were provided. | No tears in the final cohort (patients with secondary meniscus injuries were excluded) | MFC: MF1 (anterior) 16/18 (88.9 %; Level I: 9/18 [50.0 %]; II: 6/18 [33.3 %]; III: 1/18 [5.6 %]), MF2 (middle) 16/18 (88.9 %; I: 13/18 [72.2 %]; II: 3/18 [16.7 %]), MF3 (posterior) 7/18 (38.9 %; I: 7/18 [38.9 %])  LFC: LF1 (anterior)  14/18 (77.8 %; I: 10/18 [55.6 %]; II: 4/18 [22.2 %]) , LF2 (middle) 15/18 (83.3 %; I: 8/18 [44.4 %]; II: 6/18 [33.3 %]; III: 1/18 [5.6 %]), LF3 (posterior) 10/18 (55.6 %; I: 8/18 [44.4 %]; II: 2/18 [11.1 %])  MTP: MT1 (anterior) 14/18 (77.8 %; I: 13/18 [72.2 %]; II: 1/18 [5.6 %]),  MT2 (middle)  13/18 (72.2 %; I: 13/18 [72.2 %]), MT3 (posterior) 17/18 (94.4 %; I: 7/18 [38.9 %]; II: 9/18 [50.0 %]; IV: 1/18 [5.6 %])  LTP:LT1 (anterior) 12/18 (66.7 %; I: 11/18 [61.1 %]; II: 1/18 [5.6 %]),  LT2 (middle) 13/18 (72.2 %; I: 12/18 [66.7 %]; II: 1/18 [5.6 %]),  LT3 (posterior) 16/18 (88.9 %; I: 7/18 [38.9 %]; II: 4/18 [22.2 %]; III: 5/18 [27.8 %]) | NR | NR | Statistical parametric mapping in tibial internal rotation showed no significant difference in the T values between chronic and acute ACLD knees. | NR | NR |
| Crook et al | Cartilage damage | N | Y (within subject) | Healthy contralateral knee | 3.67 years, (87 days - 11 years) | NR | NR | Greatest cartilage strains found in medial femoral condyle adjacent to intercondylar notch, 6% vs 2% in ACLD vs healthy knees, p = 0.010).  Elevated cartilage strain (4% vs. 3%, p=0.01) in ACL-deficient knees | NR | NR | NR | NR | NR |
| Ates and Ogul | ALL appearance | Y | Y (chronic ACL vs acute ACL, and ALL) | Acute ACL tear | Acute: MRI at time of injury; Chronic: 2 MRIs (at injury + ≤ 30 days pre-op) | NR | NR | NR | NR | ALL status measured by muramatsu grading A-C. In the acute group (n=31, TFI = <6months), Muramatsu grade A had 10 patients (25.6%), grade B had 15 patients (38.4%), and grade C had 14 patients (35.9%). In the chronic cohort (n=61, TFI >6 months), there were only 4 grade A knees (6.5%), while there were 28 grade B knees (45.9%) and 29 grade C knees (47.5%). | NR | NR | NR |
| McDonald et al | Anterior tibial subluxation, Meniscal tears, Cartilage injury, ALL integrity | N | Y | Healthy controls, Acute ACL tears | Acute: < 2 months -Chronic: >12 months | NR | 67.5% of chronic ACL injury patients (TFI = 102.45 months) had MM tears, while 49% of acute ACL injury patients (TFI= 1.10 months) had MM tears  43% rate of LM tears in acute ACLD patients, and a 45% LM tear rate in chronic ACLD patients. | Acute ACLD patients (TFI = 1.1 months), presented with a 15% (11/74) rate of Medial Femoral Condyle (MFC) lesions, while chronic ACLD patients (TFI = 102.45 months) presented with 67.5% (27/40) MFC cartilage lesions (p= 0.001). In terms of the Lateral Femoral Condyle (LFC), the acute group had 92% (68/74) rate of lesion, while the chronic group had a 95% (38/40) lesion rate. | NR | ALL Intact:Injured  Acute ACLt, 8:48.  Chronic ACLt, 8:26  Failed ACLt, 8:56 | acute ACLD cohort (TFI =0.575 months) had a 0.11mm (± 2.55, Range: –7.2 to 6.0) anterior medial tibial subluxation (AMTS), while the chronic group (TFI = 102.45 months) had a 0.86mm (±2.39, Range: –6.0 to 7.6) AMTS. The intact ACL control group had mean -0.92mm (± 2.07, Range: –4.8 to 3.2) AMTS, which was significantly different from the chronic ACL groups (p = 0.0027). The acute ACLD cohort (TFI =0.575 months) had a 2.81 mm (± 3.20, Range: –4.8 to 9.2) anterior lateral tibial subluxation (ALTS). The chronic group (TFI = 102.45 months) had a 3.64mm (± 3.92, Range: -4.2 to 13.0) ALTS while the intact ACL control group had a -0.78mm (± 2.66, Range: –2.0 to 3.7) ALTS. Significant differences were found between the intact and chronic ACL groups (p <0.0001), and intact versus acute ACL group (p <0.0001).  the presence of a combined medial and lateral chondral defect was predictive of a mean increase in tibial subluxation of 1.941 mm (P = .0106)  medial and lateral meniscal tear was an independent predictor of lateral tibial subluxation (1.611 mm; P = .0022) | NR | NR |
| Osaki et al | Cartilage T1ρ values (degeneration) Meniscal tears | N | Y | Healthy controls, Acute ACL tears | Group A: 44.1 ± 16.0 days ( 0–76 days)  Group B: 256.3 ± 171.2 days ( 97–551days)  Group C: 1328.7 ± 397.2 days ( 744–1738 days) | NR | Medial: <12 wks: 9/28; >12 wks: 10/21.  Lateral: <12wks: 12/28; >12wks: 6/21 | MFC:  Increase T1ρ values in the anterior and middle zones of group C [Group C (>2 yr) MFC1: 50.6 ± 8.0 ms; MFC2: 52.4 ± 7.4 ms• Controls MFC1: 43.9 ± 3.3 ms; MFC2: 44.8 ± 2.2 ms]  LFC: Increased in Group C: LFC1: 46.4 ± 6.6 ms • Controls LFC1: 42.1 ± 3.0 ms  MTP, LTP: No significant differences | NR | NR | Anterior subluxation:  Group A: 5.8 ± 3.3 mm, Group B: 5.9 ± 3.1 mm, Group C: 6.5 ± 4.7 mm | NR | NR |
| Yoon et al | ACL appearance, PCL Angle (deg) | N | Y | Acute and Subacute ACL tears | **Acute**: 8.9 ± 10.7 days, **Subacute**: 60.6 ± 10.7 days. **Intermediate**: 165.5 ± 68.1 days. **Chronic**: 2166.3 ± 1599.5 days. | Type 1: increased signal strength and edematous shape.  Type 2: increased signal strength with band-like, fragmented shape.  Type 3: low signal strength and band-like/fragmented OR nonvisualized ACL.  **Acute (n=67):** Type 1 (n=55), Type 2(n=5), type 3(n=7).  **Subacute(n=19):** Type 1 (n=16), type 2(n=2), Type 3(n=1).  **Intermediate (n=23):** Type 1(n=6), Type 2(n=7), Type 3(n=9). **Chronic (n=36):** Type 1(n=6), Type 2(n=4), Type 3 (n=26).  The ratio of type 3 increased as the time to injury increased. | NR | NR | Acute: 125.1 +- 15.1 degrees,  Subacute: 116.2+- 14.1 deg,  Intermediate: 110.4 +- 15.4 deg,  Chronic: 98.7 +- 18.2 deg | NR | NR | NR | NR |
| Nawata et al | Osseous Lesions | N | Y | Acute and Subacute ACL tears | Acute: ≤1 mo; Subacute: 1–12 mo; Chronic: >12 mo | NR | NR | NR | NR | NR | NR | NR | NR |
| Yoon et al | ACL morphology, PCL angle | N | Y | Acute and subacute ACL tears | Acute: <6 weeks, Subacute: 6 weeks–3 months, Intermediate: 3 months–1 year, Chronic: >1 year | Grade 1: edematous mass-like + high signal  - Grade 2: band-like fragmented + high signal  - Grade 3: low signal or nonvisualized  High grade ACL morphology was more prevalent in older tears (p < 0.001). | NR | NR | PCL angle negatively correlated with chronicity (r = -0.567, p<0.001) | NR | NR | NR | Developed 3 logistic regression equations to classify chronicity at 6 weeks, 3 months, and 1 year |
| Liu et al | Anterior tibial subluxation, Posterior tibial slope, Meniscus tears | Y | Y | Acute ACL tears | NR | NR | reported 10.5% (4/38) rate of medial meniscus tears in acute ACLD patients (TFI = 1.1 months).21 Chronic ACLD patients with high grade pivot-shifts (TFI = 27.9 months) had a rate of medial meniscus tears of 27% (3/11), while the low grade pivot-shift group (TFI = 12.4 months) had a MM tear rate of 36.5% (15/41).  Prolonged time from injury to surgery and lateral meniscus tears are risk factors for high-grade rotatory laxity in chronic patients. | NR | NR | NR | Lateral meniscus tears were associated with high-grade rotatory instability at 6 mm or greater (p < 0.001), and at >10mm (p=0.008). | No significant differences were found in posterior tibial slope between the high-grade and low-grade groups in the chronic sub-set. | NR |
| Ye et al | Anterior tibial subluxation, Meniscal injuries, Posterior tibial slope, ALL integrity | Y | Y | NA | - High-grade: 22.8 months, Low-grade group: 11.9 months | NR | High-grade laxity ACLD patients (TFI = 22.8 months) had a 46.9% (53/113) rate of posterior horn of medial meniscus tears (PHMMT), while the low-grade group (TFI = 11.9 months) had a statistically lower PHMMT rate of 26.5% (30/113) (p = .002). Therefore, PHMMT (OR, 2.62; 95% CI, 1.35-5.08; P = .004) was determined to be an independent risk factor in high grade knee laxity.  40.7% (46/113) lateral meniscus posterior horn tear (PHLMT), while the low-grade laxity group (TFI = 11.9 months) had a 23% (26/113) PHLMT rate (p=0.004). PHLMT was determined to be an independent risk factor for knee laxity (OR, 2.39; 95% CI, 1.20-4.78; P = .014) | NR | NR | High grade knee laxity was associated with ALL abnormality (OR, 3.34; 95% CI, 1.35-8.22; P =.009) in patients with chronic ACL injury. | patients with high-grade knee laxity (TFI = 22.8 months) Patients with high-grade knee laxity had higher AMTS, measured at +2.3mm (± 2.8mm) compared to the low-grade (TFI = 11.9 months) at +0.6mm (± 2.9mm). The ALTS was also higher in the high-grade group at +6.2mm (± 3.7mm) compared to +3.5mm (±:3.3mm) in the low grade group. Both differences among the groups were statistically significant (p<0.001). | In the high-grade laxity group (n=113, TFI = 22.8 months) the lateral tibial slope was 6.4 (2.7) degrees compared to the low grade group (n=113, TFI = 11.9 months) at 5.7(2.8), (p=0.040). Across the acute and chronic groups, the medial and lateral tibial slope values did not predict laxity. | NR |
| Wang et al | Intercondylar width(ICW), Femoral condylar width(FCW) | Y | Y | Healthy controls, Acute and Subacute ACL tears | Group AR: <3 months, Group CR1: 3-12 months, Group CR2: 1-5 years, Group CR3: >5 years | NR | NR | NR | NR | NR | NR | NR | - Significant smaller ICW in group AR compared to group NI (P = 0.007). Group CR3 had an apparent but not statistically significant smaller ICW compared to other groups (P = 0.094). |
| Zhang et al | **Meniscus injuries** | Y | Y | Acute ACL tear reconstructed | Acute: median = 17 days (2-28 days),  Chronic: Median = 1077 days, (467.5 - 1784.5 days) | NR | Medial: Acute: 18/31, Chronic: 27/31. Lateral: Acute 35.5%, Chronic 51.6%.  Location: Acute: Anterior horn (3.2%), Body (12.9%), Posterior horn (22.6%); Chronic: Anterior horn (12.9%), Body (22.6%, p = 0.075), Posterior horn (38.7%, p = 0.054) | NR | NR | NR | NR | NR | NR |
| Vlychou et al | Meniscus tears, Cartilage lesions | N | N | NR | Range 3-12 months, TFI: 19.6 months | NR | Medial: 17/43(39.5%);  Lateral: 14/43 (32.6%) | 3.0 T MR imaging demonstrated 26 traumatic cartilage lesions of various grades. 11 were MFC, 11 were LFC, 2 were Patellar cartilage, and 2 were MTC among 23 patients (53.4%) and 20 patients (46.5%) with normal articular cartilage. | NR | NR | NR | NR | NR |
| Xu et al | ACL appearance, Meniscal injury, Collateral ligament injury, Tibial displacement, PCL appearance, Cartilage lesions | Y | Y | Acute ACL tear | Acute: 3.2 weeks (range: 1-47 days), Chronic: 1.3 years (range: 11 weeks - 10 years) | NR | Medial: Acute: 39/160, Chronic: 62/217  Lateral: Acute: 44/160, Chronic: 46/217 | Patellofemoral: Acute: 21/160, Chronic: 27/217, p = .018 | NR | Acute: MCL (26/160), LCL (4/160), Chronic: MCL (4/217), LCL (0/217) | Any increased distance from the posterior cortex of the lateral tibial plateau to the posterior cortex of the femur  Acute: 38/160, Chronic: 58/217 | NR | NR |
| van Meer et al | Cartilage lesions, Meniscus lesions | N | Y | Non-operative ACLt | 25.9 months (IQR, 24.7-26.9 months) | NR | NR | Lateral tibiofemoral compartment cartilage lesions:  Non-reconstruction OR: 0.53 (0.21-1.32)  Reconstruction <6 months OR:0.53 (0.21-1.32) Reconstruction >6months OR: 0.82 (0.34-2.01)  Medial tibiofemoral compartment cartilage lesions:  Non-reconstruction OR: 1  Reconstruction <6 months OR: 1.17 (0.39-3.52)  Reconstruction >6 months OR: 0.30 (0.06-1.53)  No statistical significance | NR | NR | NR | NR | Tibiofemoral osteophyte progression:  Nonoperative OR: 1  Reconstruction <6 months OR: 3.46 (0.36-33.35)  Reconstruction >6months OR: 6.07 (0.65-56.71)  No statistical significance |
| Yoo et al | Meniscal Tears | N | N | NR | First MRI: 8.5 months, Second MRI: 46.9 months | NR | Medial Meniscus, First MRI: 16/31, 2nd MRI: 26/31. Bucket Handle Tears increased from 2 to 7 from 1st to 2nd MRI. Longitudinal tears increased from 14 to 19. | NR | NR | NR | NR | NR | NR |
| Nonaka et al | ACL appearance, Meniscus lesions | Y | Y | Control group n=41,  chronic group n=50 | NR | NR | Lateral Meniscus tears: Gap-positive: 20/29 (69%) Gap-Negative:5/21(71%) | NR | NR | NR | NR | NR | NR. |
| Oronowicz et al | PCL appearance, Collateral ligament lesions, Meniscus injuries | N | Y | Acute ACL tear | Acute: 0.7 months (0.2–119.3), Chronic:12.9 months (8.1–44.4) | NR | NR | NR | The median PCL–PCA was significantly lower (− 7.7°) in chronic injuries compared to acute injuries (p < 0.01; r = 0.33) | NR | NR | NR | NR |
| Gong et al | PCL appearance, Notch appearance | Y | Y | Acute and Subacute ACL tears | Acute = <6 weeks, Subacute = 6 weeks - 6 months, chronic > 6 months | NR | NR | NR | PCL-Index:  Acute = 5.3 (1.7),  Subacute =5.1(1.6), Chronic = 4.8 (1.4). | NR | NR | NR | LFNS(mm) : Acute = 1.0 (0.6), Subacute = 1.0 (0.6). Chronic = 0.9(0.4). Insignificant differences based on time from injury (acute, subacute, chronic) |
| Mishima et al | Anterior Tibial Subluxation | N | Y | Health contralateral Knee | Entire Sample: 27 months | NR | NR | NR | NR | NR | 2.1mm (SD: 1.3, Range: 0-4.6mm) mean of anterior tibial subluxation compared to healthy contralateral knees. There was also a positive correlation in the extent of the ATS and time since injury (r =.332, P < 0.0013). Patients without ATS had a significantly shorter history of ACL deficiency than those with ATS (P < 0.031) | NR |  |

NR, not reported

**Supplementary Table 3. Pooled Analysis of Meniscal Lesions**

| **Study** | **Group** | **Sample Size** | **Time from Injury to MRI** | **Medial Meniscus Tear** | **Lateral Meniscus Tear** |
| --- | --- | --- | --- | --- | --- |
| **Dimond (1998)** | Chronic | 24 | 55.4 weeks (26.2- 515 weeks) | 19/24 | 5/24 |
| **Dimond (1998)** | Acute ACLt | 60 | 2.5 weeks (1 day to 5.7 weeks) | 14/60 | 16/60 |
| **McDonald (2017)** | Acute ACL injury | 74 | 33.4 (15.4) days | 36/74 | 32/74 |
| **McDonald (2017)** | Chronic ACL injury | 40 | 3119 (3632) days | 27/40 | 18/40 |
| **McDonald (2017)** | Failed ACLR | 72 | N/A | 52/72 | 32/72 |
| **Liu (2022)** | High-Grade Acute | 19 | 1.1 (0.6-1.6) months | 9/19 | 12/19 |
| **Liu (2022)** | Low-Grade Acute | 19 | 1.0 (0.6-1.5) months | 4/19 | 9/19 |
| **Liu (2022)** | High-Grade Chronic | 11 | 27.9 (11.9-43.9) months | 6/11 | 8/11 |
| **Liu (2022)** | Low-Grade Chronic | 41 | 12.4 (9.6-15.1) months | 20/41 | 7/41 |
| **Nonaka (2024)** | Gap positive chronic | 29 | 1,121.3 (3,089.5) days | NR | 20/29 |
| **Nonaka (2024)** | Gap negative chronic | 21 | 329.9 (778.9) days | NR | 15/21 |
| **Osaki (2015)** | Acute ACLt | 28 | 1.45 months (1.39 – 1.56 months) | 9/28 | 12/28 |
| **Osaki (2015)** | Chronic ACLt | 21 | 20.16 months (16.04 – 24.7 months) | 10/21 | 6/21 |
| **Vlychou (2011)** | Chronic ACL Injury | 43 | 19.6 months | 17/43 | 14/43 |
| **Xu (2018)** | Acute ACL Tears | 160 | 3.2 weeks (1-47 days) | 51/160 | 56/160 |
| **Xu (2018)** | Chronic ACL Tears | 217 | 1.3 years (11 weeks - 10 years) | 75/217 | 60/217 |
| **Yoo (2009)** | First MRI group | 31 | 8.45 months | 16/31 | NR |
| **Yoo (2009)** | Second MRI group | 31 | 46.9 months | 26/31 | NR |
| **Zhang (2023)** | Acute ACL Injury | 31 | 30 days | 18/31 | 11/31 |
| **Zhang (2023)** | Chronic ACL Injury | 31 | 1080 days | 27/31 | 16/31 |

NR, Not reported

**Supplementary Table 4: Search Strategy**

| **MEDLINE: 501 Studies** | | **CINAHL:256 studies** | **Embase: 626 Studies** | **Central: 37 studies** | **Total** |
| --- | --- | --- | --- | --- | --- |
| Strategy | Studies | “ | “ | “ |  |
| 1. exp Anterior Cruciate Ligament/ or exp Anterior Cruciate Ligament Injuries/ or ACL.mp. | 32299 | “ | “ | “ |  |
| 2. exp Anterior Cruciate Ligament Injuries/ or exp Rupture/ OR tear.mp. or exp tears/ OR exp Knee Injuries/ | 128853 | “ | “ | “ |  |
| 3. (chronic or 6-month).mp. | 4308860 | “ | “ | “ |  |
| 4. exp Magnetic Resonance Imaging/ OR MRI.mp. | 1347348 | “ | “ | “ |  |
| 1 AND 2 AND 3 AND 4 | 502 | 256 | 626 | 37 | 1421 studies |

“ All search strategies teams from the MEDLINE search were translated to CINAHL, EMBASE and CENTRAL search terms with the correct syntax for each respective database, 1421 studies returned 563 duplicates. 858 studies were screened for title and abstract, with 702 being irrelevant. 156 studies underwent full-text screening, with 134 being excluded (mainly for wrong study designs, populations, no chronic groups, or unavailable full texts).

**Supplementary Table 5: PRISMA Checklist**

| **Section and Topic** | **Item #** | **Checklist item** | **Location where item is reported** |
| --- | --- | --- | --- |
| **TITLE** | | |  |
| Title | 1 | Identify the report as a systematic review. | Title Page |
| **ABSTRACT** | | |  |
| Abstract | 2 | See the PRISMA 2020 for Abstracts checklist. | Abstract |
| **INTRODUCTION** | | |  |
| Rationale | 3 | Describe the rationale for the review in the context of existing knowledge. | Line 57-61 |
| Objectives | 4 | Provide an explicit statement of the objective(s) or question(s) the review addresses. | Line 57-61 |
| **METHODS** | | |  |
| Eligibility criteria | 5 | Specify the inclusion and exclusion criteria for the review and how studies were grouped for the syntheses. | Methods |
| Information sources | 6 | Specify all databases, registers, websites, organisations, reference lists and other sources searched or consulted to identify studies. Specify the date when each source was last searched or consulted. | Line 64 |
| Search strategy | 7 | Present the full search strategies for all databases, registers and websites, including any filters and limits used. | Line 64-67 |
| Selection process | 8 | Specify the methods used to decide whether a study met the inclusion criteria of the review, including how many reviewers screened each record and each report retrieved, whether they worked independently, and if applicable, details of automation tools used in the process. | Lines 71-75 |
| Data collection process | 9 | Specify the methods used to collect data from reports, including how many reviewers collected data from each report, whether they worked independently, any processes for obtaining or confirming data from study investigators, and if applicable, details of automation tools used in the process. | Lines 83-88 |
| Data items | 10a | List and define all outcomes for which data were sought. Specify whether all results that were compatible with each outcome domain in each study were sought (e.g. for all measures, time points, analyses), and if not, the methods used to decide which results to collect. | Lines 100-5 |
|  | 10b | List and define all other variables for which data were sought (e.g. participant and intervention characteristics, funding sources). Describe any assumptions made about any missing or unclear information. | Lines 100-5 |
| Study risk of bias assessment | 11 | Specify the methods used to assess risk of bias in the included studies, including details of the tool(s) used, how many reviewers assessed each study and whether they worked independently, and if applicable, details of automation tools used in the process. | Lines 90-3 |
| Effect measures | 12 | Specify for each outcome the effect measure(s) (e.g. risk ratio, mean difference) used in the synthesis or presentation of results. | Lines 101-8 |
| Synthesis methods | 13a | Describe the processes used to decide which studies were eligible for each synthesis (e.g. tabulating the study intervention characteristics and comparing against the planned groups for each synthesis (item #5)). | Lines 101-8 |
|  | 13b | Describe any methods required to prepare the data for presentation or synthesis, such as handling of missing summary statistics, or data conversions. | Lines 101-8 |
|  | 13c | Describe any methods used to tabulate or visually display results of individual studies and syntheses. | Lines 101-8 |
|  | 13d | Describe any methods used to synthesize results and provide a rationale for the choice(s). If meta-analysis was performed, describe the model(s), method(s) to identify the presence and extent of statistical heterogeneity, and software package(s) used. | Lines 101-8 |
|  | 13e | Describe any methods used to explore possible causes of heterogeneity among study results (e.g. subgroup analysis, meta-regression). | None |
|  | 13f | Describe any sensitivity analyses conducted to assess robustness of the synthesized results. | None |
| Reporting bias assessment | 14 | Describe any methods used to assess risk of bias due to missing results in a synthesis (arising from reporting biases). | Supplementary table 3 |
| Certainty assessment | 15 | Describe any methods used to assess certainty (or confidence) in the body of evidence for an outcome. | None |
| **RESULTS** | | |  |
| Study selection | 16a | Describe the results of the search and selection process, from the number of records identified in the search to the number of studies included in the review, ideally using a flow diagram. | Lines 111-115 |
|  | 16b | Cite studies that might appear to meet the inclusion criteria, but which were excluded, and explain why they were excluded. | Figure 1 |
| Study characteristics | 17 | Cite each included study and present its characteristics. | 117-128 |
| Risk of bias in studies | 18 | Present assessments of risk of bias for each included study. | Supplementary Table 1 |
| Results of individual studies | 19 | For all outcomes, present, for each study: (a) summary statistics for each group (where appropriate) and (b) an effect estimate and its precision (e.g. confidence/credible interval), ideally using structured tables or plots. | Table 2 |
| Results of syntheses | 20a | For each synthesis, briefly summarise the characteristics and risk of bias among contributing studies. | Supplementary Table 1 |
|  | 20b | Present results of all statistical syntheses conducted. If meta-analysis was done, present for each the summary estimate and its precision (e.g. confidence/credible interval) and measures of statistical heterogeneity. If comparing groups, describe the direction of the effect. | Figures 1-5 |
|  | 20c | Present results of all investigations of possible causes of heterogeneity among study results. | Figures 1-5 |
|  | 20d | Present results of all sensitivity analyses conducted to assess the robustness of the synthesized results. | Figures 1-5 |
| Reporting biases | 21 | Present assessments of risk of bias due to missing results (arising from reporting biases) for each synthesis assessed. | None |
| Certainty of evidence | 22 | Present assessments of certainty (or confidence) in the body of evidence for each outcome assessed. | None |
| **DISCUSSION** | | |  |
| Discussion | 23a | Provide a general interpretation of the results in the context of other evidence. | Discussion |
|  | 23b | Discuss any limitations of the evidence included in the review. | Limitations |
|  | 23c | Discuss any limitations of the review processes used. | Limitations |
|  | 23d | Discuss implications of the results for practice, policy, and future research. | Conclusion |
| **OTHER INFORMATION** | | |  |
| Registration and protocol | 24a | Provide registration information for the review, including register name and registration number, or state that the review was not registered. | PROSPERO |
|  | 24b | Indicate where the review protocol can be accessed, or state that a protocol was not prepared. | Not Prepared |
|  | 24c | Describe and explain any amendments to information provided at registration or in the protocol. | Authorship updated |
| Support | 25 | Describe sources of financial or non-financial support for the review, and the role of the funders or sponsors in the review. | None |
| Competing interests | 26 | Declare any competing interests of review authors. | None |
| Availability of data, code and other materials | 27 | Report which of the following are publicly available and where they can be found: template data collection forms; data extracted from included studies; data used for all analyses; analytic code; any other materials used in the review. | None |
